# Genome-Wide Association for Abdominal Subcutaneous and Visceral Adipose Reveals a Novel Locus for Visceral Fat in Women

**DOI:** 10.1371/journal.pgen.1002695

**Published:** 2012-05-10

**Authors:** Caroline S. Fox, Yongmei Liu, Charles C. White, Mary Feitosa, Albert V. Smith, Nancy Heard-Costa, Kurt Lohman, GIANT Consortium, MAGIC Consortium, GLGC Consortium, Andrew D. Johnson, Meredith C. Foster, Danielle M. Greenawalt, Paula Griffin, Jinghong Ding, Anne B. Newman, Fran Tylavsky, Iva Miljkovic, Stephen B. Kritchevsky, Lenore Launer, Melissa Garcia, Gudny Eiriksdottir, J. Jeffrey Carr, Vilmunder Gudnason, Tamara B. Harris, L. Adrienne Cupples, Ingrid B. Borecki

**Affiliations:** 1 Framingham Heart Study, National Heart, Lung, and Blood Institute (NHLBI), National Institutes of Health, Framingham, Massachusetts, United States of America; 2 Center for Population Studies, National Heart, Lung, and Blood Institute (NHLBI), National Institutes of Health, Framingham, Massachusetts, United States of America; 3 Division of Endocrinology, Brigham and Women's Hospital and Harvard Medical School, Boston, Massachusetts, United States of America; 4 Department of Epidemiology and Prevention, Public Health Sciences, Wake Forest School of Medicine, Winston-Salem, North Carolina, United States of America; 5 Department of Biostatistics, Boston University School of Public Health, Boston, Massachusetts, United States of America; 6 Division of Statistical Genomics, Department of Genetics, Washington University School of Medicine, St. Louis, Missouri, United States of America; 7 Icelandic Heart Association, Research Institute, Kopavogur, Iceland; 8 University of Iceland, Reykjavik, Iceland; 9 Merck Research Laboratories, Boston, Massachusetts, United States of America; 10 Department of Internal Medicine/Geriatrics, Wake Forest School of Medicine, Winston-Salem, North Carolina, United States of America; 11 Department of Epidemiology, University of Pittsburgh, Pittsburgh, Pennsylvania, United States of America; 12 Department of Preventive Medicine, University of Tennessee, Memphis, Tennessee, United States of America; 13 Center for Aging and Population Health, Department of Epidemiology, University of Pittsburgh, Pittsburgh, Pennsylvania, United States of America; 14 Laboratory of Epidemiology, Demography, and Biometry, National Institute on Aging, National Institutes of Health, Bethesda, Maryland, United States of America; 15 Departments of Radiologic Sciences, Internal Medicine-Cardiology, and Public Health Sciences, Wake Forest University School of Medicine, Winston-Salem, North Carolina, United States of America; University of Alabama at Birmingham, United States of America

## Abstract

Body fat distribution, particularly centralized obesity, is associated with metabolic risk above and beyond total adiposity. We performed genome-wide association of abdominal adipose depots quantified using computed tomography (CT) to uncover novel loci for body fat distribution among participants of European ancestry. Subcutaneous and visceral fat were quantified in 5,560 women and 4,997 men from 4 population-based studies. Genome-wide genotyping was performed using standard arrays and imputed to ∼2.5 million Hapmap SNPs. Each study performed a genome-wide association analysis of subcutaneous adipose tissue (SAT), visceral adipose tissue (VAT), VAT adjusted for body mass index, and VAT/SAT ratio (a metric of the propensity to store fat viscerally as compared to subcutaneously) in the overall sample and in women and men separately. A weighted z-score meta-analysis was conducted. For the VAT/SAT ratio, our most significant p-value was rs11118316 at *LYPLAL1* gene (p = 3.1×10E-09), previously identified in association with waist–hip ratio. For SAT, the most significant SNP was in the *FTO* gene (p = 5.9×10E-08). Given the known gender differences in body fat distribution, we performed sex-specific analyses. Our most significant finding was for VAT in women, rs1659258 near *THNSL2* (p = 1.6×10-08), but not men (p = 0.75). Validation of this SNP in the GIANT consortium data demonstrated a similar sex-specific pattern, with observed significance in women (p = 0.006) but not men (p = 0.24) for BMI and waist circumference (p = 0.04 [women], p = 0.49 [men]). Finally, we interrogated our data for the 14 recently published loci for body fat distribution (measured by waist–hip ratio adjusted for BMI); associations were observed at 7 of these loci. In contrast, we observed associations at only 7/32 loci previously identified in association with BMI; the majority of overlap was observed with SAT. Genome-wide association for visceral and subcutaneous fat revealed a SNP for VAT in women. More refined phenotypes for body composition and fat distribution can detect new loci not previously uncovered in large-scale GWAS of anthropometric traits.

## Author Summary

Body fat distribution, particularly centralized obesity, is associated with metabolic risk above and beyond total adiposity. We performed genome-wide association of abdominal adipose depots quantified using computed tomography (CT) to uncover novel loci for body fat distribution among participants of European ancestry. We quantified subcutaneous and visceral fat in more than 10,000 women and men who also had genome-wide association data available. Given the known gender differences in body fat distribution, we performed sex-specific analyses. Our most significant finding was for VAT in women, near the *THNSL2* gene. These findings were not observed in men. We also interrogated our data for the 14 recently published loci for body fat distribution (measured by waist–hip ratio adjusted for BMI); associations were observed for 7 of these loci, most notably for VAT/SAT ratio. We conclude that genome-wide association for visceral and subcutaneous fat revealed a SNP for VAT in women. More refined phenotypes for body composition and fat distribution can detect new loci not uncovered in large-scale GWAS of anthropometric traits.

## Introduction

Obesity is an important risk factor for cardiometabolic outcomes [1]–[5]. Heterogeneity in the regional deposition of fat, particularly, visceral adipose tissue (VAT), may be more deleterious than total body obesity. Numerous epidemiologic studies have demonstrated that central obesity, measured by simple anthropometric measures including waist circumference or waist-hip-ratio (WHR), is associated with cardiovascular disease (CVD) and glucose, insulin, and lipid metabolism, independent of overall obesity as measured by body mass index (BMI) [6]–[19]. However, waist circumference is limited due to its inability to discriminate between VAT and subcutaneous adipose tissue (SAT) [20]. Computed tomography (CT) provides a more direct and precise assessment of adipose tissue compartments. In many studies, the associations between CVD risk factors and directly-measured VAT are stronger than the associations observed with other typical anthropometric measures [21]–[28].

Prior studies have shown that indices of body fat distribution, including waist circumference, VAT, and SAT are heritable [20], [29]–[31]. A recent large-scale genome-wide association study (GWAS) identified 14 loci in association with waist-hip-ratio [32], providing proof-of-principle for the concept that genetic variants are associated with body fat distribution above and beyond generalized adiposity. However, there are currently no large-scale GWAS for directly-measured VAT and SAT. Thus, the purpose of the present study was to perform GWAS for VAT and SAT in 4 large population-based cohorts. We analyzed SAT, VAT, VAT adjusted for BMI, and the VAT/SAT ratio, a metric of the propensity to store fat viscerally as compared to subcutaneously. Given the known sex differences in body fat distribution [33], we additionally performed sex-specific analyses.

## Results

The characteristics of the study sample are presented in Table 1 and Table S1. Overall, 5560 women and 4997 men were available for analysis. Study participants ranged in age from their thirties to their mid seventies, and BMI ranged from 26.6 kg/m^2^ to 28.8 kg/m^2^.

**Table 1:** Study Sample Characteristics, VATGen Consortium.

Study	SAT n	VAT n	Women % (n)	Age (years)	BMI (kg/m2)	SAT (cm2 or cm3)*	VAT (cm2 or cm3)*	VATSAT Ratio
Fram Overall	3158	3158	48.01 (1516)	52.8 (11.9)	27.8(5.2)	2875(1379)	1822(1033)	0.69(0.39)
Fram Women	1516	1516	100	54.2(11.3)	27.0(5.8)	3135(1514)	1364(833)	0.45( 0.21)
Fram Men	1642	1642	0	51.5(12.2)	28.4(4.5)	2636(1193)	2244(1020)	0.91( 0.39)
FamHS Overall	2659	2659	55.28 (1470)	57.2 (13.3)	28.8(5.7)	286(132)	167(91)	0.65(0.39)
FamHS Women	1470	1470	100	57.6(13.1)	28.5(6.3)	315(142)	138(78)	0.46( 0.25)
FamHS Men	1189	1189	0	56.7(13.4)	29.3(4.7)	249(108)	203(94)	0.87( 0.40)
HABC overall	1568	1568	47.1 (739)	73.8 (2.8)	26.6 (4.1)	266.3 (101.9)	153.4(69.5)	0.63(0.33)
HABC women	739	739	100	73.7 (2.8)	26.1 (4.4)	311.3 (104.9)	134.4 (62.0)	0.45 (0.19)
HABC men	829	829	0	73.9 (2.9)	27.1 (3.7)	226.1 (80.1)	170.3 (71.6)	0.79 (0.34)
AGES overall	3172	3172	57.85 (1835)	76.41 (.49)	27.1 (4.4)	257.5 (113.2)	172.7 (80.7)	0.77 (.44)
AGES women	1835	1835	100 (1835)	76.34 (0)	27.2 (4.8)	296.1 (115.2)	149.2 (66.9)	0.54 (.27)
AGES men	1337	1337	0 (0)	76.51 (0)	26.9 (3.8)	204.6 (85.8)	205.1 (86.5)	1.08 (.44)

Data shown as mean (standard deviation) unless otherwise indicated.

*: cm3 for the Framingham Heart Study; all other studies are measured in cm2.

### Heritability Analyses

In order to document a genetic or familial component of directly-imaged CT adipose tissue traits, we previously performed heritability analyses of VAT (*h2* 36%) and SAT (*h2* 57%) [20]. For the present analysis, we additionally calculated the heritability of VAT and SAT in the Family Heart Study and the VAT/SAT ratio in both the Family Heart Study and the Framingham Heart Study. In the Family Heart Study, the heritability of VAT and SAT was 36% and 44%, respectively. The heritability of the VAT/SAT ratio was 43%; after adjustment for either BMI or VAT, the heritability was not materially different (44% and 47%, respectively). Similarly, we found that the heritability of the VAT/SAT ratio was 55% (p<0.0001) in the Framingham Heart Study, which was essentially unchanged after adjustment for BMI (h^2^ 55%) or VAT (64%).

### Stage 1 Discovery Results

After confirming a heritable component to directly imaged CT adipose tissue traits, we proceeded with GWAS. To assess for occult population stratification, we examined q-q plots for all traits (SAT, VAT, VAT-adjusted-for-BMI, and VAT/SAT ratio in the overall sample and in women and men separately), which can be found in Figure S1. All lambda values were <1.08, with little evidence to suggest unaccounted for population stratification. Manhattan plots for these traits can be found in Figure S2, with p-values<5.0*10E08 for the VAT/SAT ratio overall and VAT in women.

**Figure 1:**
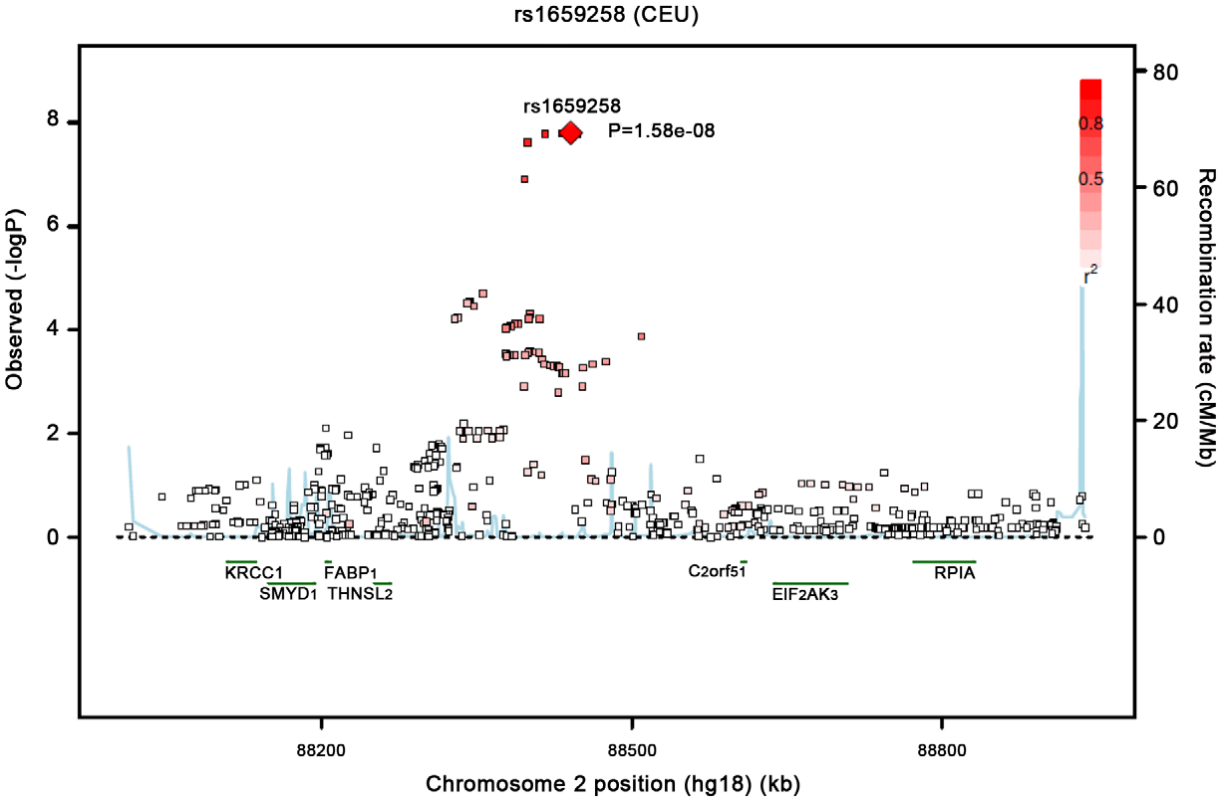
Regional Association Plot of the Chromosome 2 region for VAT in women.

**Figure 2:**
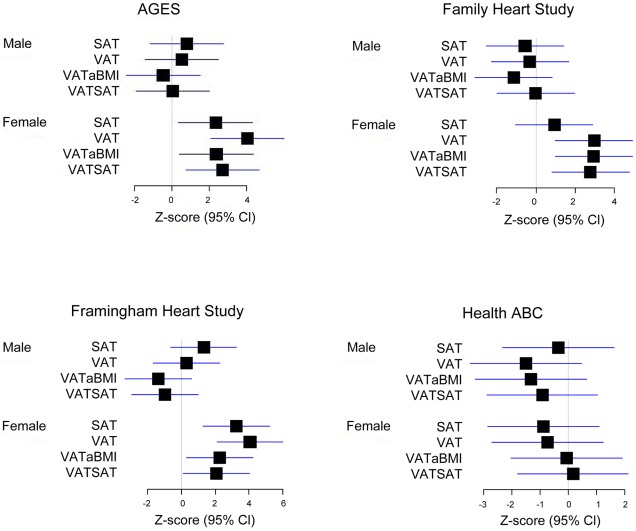
Association of rs1659258 in all 4 discovery cohorts. Results are shown modeled per copy of the trait-increasing A allele. Within each study, data presented represent the beta coefficient indexed to the standard error. Bars represent 95% confidence intervals. VATSAT is the VAT/SAT ratio, and VATaBMI is VAT-adjusted-for-BMI.

Our most significant finding was for rs11118316 at *LYPLAL1* for the VAT/SAT ratio (Table S2). This SNP is in low to moderate LD with rs4846567 (r^2^ = 0.285, D′ 0.935), which was previously identified in the GIANT consortium in association with WHR-adjusted-for-BMI [32]. It is notable that in the GIANT consortium, rs4846567 was only associated with WHR in women (p = 4.9*10E-33) but not men (p = 0.36), whereas rs11118316 was associated with both women (p = 4.5*10E-6) and men (p = 8.3*10E-5) in the present analysis. We further note that for rs4846567, the p-value is 4.4*10E-04 in women but p = 0.05 in men. Thus, it is possible that we have identified a slightly different locus with varying sex differences. Our next genome-wide significant finding was for rs1659258 at chromosome 2 for VAT in women (p = 1.58*10E-08; Table S2 and Figure 1). Imputation scores for this SNP ranged from 0.98 to 1.0. This region has not previously been identified in association with adiposity phenotypes. Table 2 shows the results for rs1659258 across the abdominal adiposity traits in our meta-analysis. We observed no association in men (p = 0.75) for VAT. In women, we observed a nominally-significant signal for SAT in women (p = 0.002), and for VAT-adjusted-for-BMI (p = 6.9*10E-05). Figure 2 shows the standardized beta coefficients in women as compared to men for all traits in each contributing study.

**Table 2:** Results of rs1659258 in the VATGen meta-analysis; results modeled per copy of the trait-increasing A allele and for independent validation in the GIANT Consortium (non-overlapping studies).*

Trait	Effect Direction	P-value
VAT women	+	1.6E-08
VAT overall	+	0.001
VAT men	−	0.75
VATaBMI women	+	6.9E-05
VATaBMI overall	+	0.52
VATaBMI men	−	0.03
VATSAT women	+	5.1E-05
VATSAT overall	+	0.03
VATSAT men	−	0.36
SAT women	+	0.002
SAT overall	+	0.006
SAT men	+	0.46
**Independent Validation, GIANT**		
BMI men	+	0.24
BMI women	+	0.006
WC men	+	0.49
WC women	+	0.04

*: GIANT sample sizes for women and men are as follows: BMI (58208, 49092); WC (39471, 31406).

Given our finding of a stronger association in women as compared to men at rs1659258 for VAT, we formally tested a sex interaction term, and found a significant effect in the magnitude of the association between our lead SNP and VAT in women as compared to men (p_interaction_ = 0.0002).

All additional independent SNPs with p-values<9.9*10E-06 can be found in Table S2.

### Stage 2 Validation

We focused on rs1659258 as a new locus for independent validation in the non-overlapping cohorts that are part of the GIANT consortium data. Results are displayed in Table 2, with evidence for replication at this locus in women (p = 0.006) but not men (p = 0.24) for BMI. Similar results were observed for waist circumference.

### Metabolic Traits Association Studies with rs1659258

After confirming the association of rs1659258 with measures of adiposity in a sex-specific manner, we next sought to perform association studies with correlated metabolic traits. Because VAT is associated with glycemic and lipid traits [20], [34]–[37], we requested sex-specific associations of rs1659258 in the Global Lipids Genetics Consortium [38] for lipid traits and in the MAGIC consortium for traits related to glucose metabolism. Overall, we observed a nominal and direction-consistent association (i.e., same allele associated with higher VAT and lower HDL) with HDL (p = 0.019, n = 98,263), but no robust associations for LDL, triglycerides, or total cholesterol (all p>0.06), which is surprising given the phenotypic correlations between VAT and triglycerides [20]. In sex-specific analyses, we observed no association for this SNP with HDL, triglycerides, or total cholesterol for either women (all p>0.09, personal communication) or men (all p>0.73, personal communication). In MAGIC, we observed a direction-consistent and borderline statistically significant result for this SNP in women (p = 0.048, n = 43,754) but not men (p = 0.39, n = 36,514) for fasting glucose (personal communication, MAGIC consortium).

### Test of Age Interaction with rs1659258

Because fat distribution can vary by age, we performed a formal age interaction test between rs1659258 and VAT in women only. Stratifying above and below age 60 years, we did not observe a significant sex interaction (p = 0.79).

### Interrogation of Published Loci for WHR and BMI Loci

In order to understand whether directly-imaged adipose traits are associated with previously-identified loci in a GWAS for fat distribution using anthropometric traits, we performed an association analysis of the 14 previously-published loci for WHR-adjusted-for-BMI for all 4 of our traits in the overall sample and by sex (Table 3) in the results from the GIANT consortium [32]. Overall, we observed associations (defined as p<0.01) for 7/14 of the previously reported loci, most of which were direction-consistent (with the exception of loci associated with SAT, consistent with the fact that SAT and hip are highly correlated traits [r = 0.92 among 2656 individuals from the Family Heart Study]). We observed only one association with VAT at the *NISCH-STAB1* locus.

**Table 3:** Association of SNPs from a Recently Published GWAS of Body Fat Distribution* (Heid IM et al, NG, 2010) [32].

					VATSAT		VAT		SAT		VATaBMI	
Trait	SNP	Chr	Nearby Genes	Coded Allele	Z statistic	P-value	Z statistic	P-value	Z statistic	P-value	Z statistic	P-value
Men	rs984222	1	*TBX15-WARS2*	G	+	0.13	−	0.87	−	0.09	+	0.18
	rs1011731	1	*DNM3-PIGC*	G	+	0.11	+	0.67	−	0.48	+	0.50
	rs4846567	1	*LYPLAL1*	G	+	0.05	−	0.66	−	0.03	−	0.66
	rs10195252	2	*GRB14*	T	+	0.01	−	0.20	−	0.0002	+	0.60
	rs6784615	3	*NISCH-STAB1*	T	+	0.26	+	0.06	+	0.82	+	0.01
	rs6795735	3	*ADAMTS9*	C	+	0.27	−	0.30	−	0.008	+	0.87
	rs6861681	5	*CPEB4*	A	+	0.06	+	0.55	−	0.31	+	0.19
	rs1294421	6	*LY86*	G	+	0.02	+	0.11	+	0.82	+	0.21
	rs6905288	6	*VEGFA*	A	+	0.03	+	0.98	−	0.14	+	0.69
	rs9491696	6	*RSPO3*	G	−	0.67	−	0.47	−	0.56	−	0.66
	rs1055144	7	*NFE2L3*	T	+	0.95	+	0.12	+	0.30	+	0.43
	rs718314	12	*ITPR2-SSPN*	G	+	0.29	−	0.54	−	0.01	+	0.62
	rs1443512	12	*HOXC13*	A	−	0.06	+	0.45	+	0.05	−	0.81
	rs4823006	22	*ZNRF3-KREMEN1*	A	+	0.24	−	0.76	−	0.10	+	0.94
Women	rs984222	1	*TBX15-WARS2*	G	+	0.91	+	0.63	+	0.82	+	0.10
	rs1011731	1	*DNM3-PIGC*	G	+	0.32	+	0.04	+	0.40	+	0.07
	rs4846567	1	*LYPLAL1*	G	+	0.0004	+	0.10	−	0.06	+	0.0009
	rs10195252	2	*GRB14*	T	+	0.43	−	0.86	−	0.54	+	0.60
	rs6784615	3	*NISCH-STAB1*	T	−	0.77	−	0.008	−	0.10	−	0.11
	rs6795735	3	*ADAMTS9*	C	+	0.01	+	0.20	−	0.17	+	0.02
	rs6861681	5	*CPEB4*	A	+	0.40	+	0.20	+	0.44	+	0.08
	rs1294421	6	*LY86*	G	+	0.05	+	0.14	+	0.72	+	0.18
	rs6905288	6	*VEGFA*	A	+	0.54	+	0.95	−	0.36	+	0.08
	rs9491696	6	*RSPO3*	G	+	0.005	+	0.34	−	0.23	+	0.02
	rs1055144	7	*NFE2L3*	T	+	0.17	+	0.77	−	0.88	+	0.41
	rs718314	12	*ITPR2-SSPN*	G	+	0.17	+	0.32	−	0.43	+	0.009
	rs1443512	12	*HOXC13*	A	+	0.32	+	0.49	+	0.99	+	*0.046*
	rs4823006	22	*ZNRF3-KREMEN1*	A	+	0.07	+	0.25	+	0.46	+	0.22
Overall	rs984222	1	*TBX15-WARS2*	G	+	0.25	+	0.80	−	0.47	+	0.08
	rs1011731	1	*DNM3-PIGC*	G	+	0.07	+	0.09	+	0.91	+	0.12
	rs4846567	1	*LYPLAL1*	G	+	0.0002	+	0.47	−	0.01	+	0.15
	rs10195252	2	*GRB14*	T	+	0.03	−	0.28	−	0.01	+	0.58
	rs6784615	3	*NISCH-STAB1*	T	+	0.66	−	0.60	−	0.22	+	0.37
	rs6795735	3	*ADAMTS9*	C	+	0.01	+	0.95	−	0.005	+	0.09
	rs6861681	5	*CPEB4*	A	+	0.05	+	0.19	−	0.92	+	0.03
	rs1294421	6	*LY86*	G	+	0.003	+	0.05	−	0.89	+	0.06
	rs6905288	6	*VEGFA*	A	+	0.05	−	0.98	−	0.06	+	0.15
	rs9491696	6	*RSPO3*	G	+	0.13	−	0.94	−	0.11	+	0.35
	rs1055144	7	*NFE2L3*	T	+	0.33	+	0.22	+	0.64	+	0.30
	rs718314	12	*ITPR2-SSPN*	G	+	0.07	+	0.70	−	0.05	+	0.04
	rs1443512	12	*HOXC13*	A	−	0.51	+	0.45	+	0.32	+	0.36
	rs4823006	22	*ZNRF3-KREMEN1*	A	+	0.06	+	0.77	−	0.67	+	0.72

All data modeled relative to the previously-published trait-increasing allele; the z-statistic indicates the effect direction relative to the coded allele.

*: Measured by WHR-adjusted-for-BMI.

We additionally performed an association analysis of all of the 32 published BMI loci identified via the GIANT consortium (Table 4) [39]. Overall, we observed associations (defined as p<0.01) at 7/32 loci, the majority of which overlapped for SAT and were all direction-consistent with the exception of VAT/SAT ratio at *NEGR1*. We observed very few associations with VAT, with the exception of *FTO* and *NRXN3*.

**Table 4:** Association of validated SNPs for BMI (from Speliotes et al, Nature Genetics 2010) [39].

					VATSAT		VAT		SAT		VATaBMI	
	SNP	Chr	Closest Gene	Coded allele	Z statistic	P-value	Z statistic	P-value	Z statistic	P-value	Z statistic	P-value
Men	rs9816226	3	*ETV5*	T	−	0.06	+	0.74	+	0.08	−	0.28
	rs2287019	19	*GIPR*	C	−	0.34	+	0.27	+	0.03	−	0.50
	rs1558902	16	*FTO*	A	−	0.21	+	0.007	+	9.7E-05	+	0.82
	rs11847697	14	*PRKD1*	T	−	0.84	−	0.87	+	0.83	−	0.76
	rs7359397	16	*SH2B1*	T	+	0.20	+	0.03	+	0.10	+	0.25
	rs2241423	15	*MAP2K5*	G	−	0.36	−	0.16	+	0.62	−	0.11
	rs7138803	12	*FAIM2*	A	−	0.08	−	0.37	+	0.47	−	0.28
	rs10150332	14	*NRXN3*	C	+	0.71	+	0.008	+	0.003	+	0.30
	rs12444979	16	*GPRC5B*	C	−	0.15	+	0.40	+	0.08	−	0.46
	rs887912	2	*FANCL*	T	+	0.23	+	0.06	+	0.25	+	0.46
	rs2112347	5	*FLJ35779*	T	+	0.54	+	0.09	+	0.25	+	0.24
	rs2815752	1	*NEGR1*	A	−	0.003	+	0.05	+	3.9E-06	−	0.30
	rs4836133	5	*ZNF608*	A	−	0.29	+	0.52	+	0.10	−	0.31
	rs571312	18	*MC4R*	A	−	0.50	+	0.67	+	0.41	−	0.48
	rs3810291	19	*TMEM160*	A	−	0.16	+	0.41	+	0.03	−	0.47
	rs4929949	11	*RPL27A*	C	−	0.03	+	0.81	+	0.07	−	0.29
	rs713586	2	*RBJ, ADCY3*	C	−	0.43	−	0.61	−	0.85	−	0.87
	rs2890652	2	*LRP1B*	C	−	0.12	−	0.13	−	0.98	−	0.03
	rs987237	6	*TFAP2B*	G	−	0.21	+	0.47	+	0.08	−	0.28
	rs10968576	9	*LINGO2*	G	−	0.53	+	0.56	+	0.25	−	0.80
	rs13107325	4	*SLC39A8*	T	+	0.42	+	0.78	−	0.72	−	0.72
	rs3817334	11	*MTCH2*	T	+	0.79	+	0.69	+	0.32	−	0.90
	rs206936	6	*NUDT3*	G	−	0.95	−	0.91	+	0.87	−	0.33
	rs2867125	2	*TMEM18*	C	−	0.10	−	0.49	+	0.12	−	0.40
	rs543874	1	*SEC16B*	G	−	0.34	+	0.49	+	0.09	−	0.60
	rs1555543	1	*PTBP2*	C	+	0.32	+	0.38	+	0.79	+	0.35
	rs1514175	1	*TNNI3K*	A	−	0.15	−	0.11	−	0.80	−	0.17
	rs10938397	4	*GNPDA2*	G	−	0.04	−	0.55	+	0.18	−	0.15
	rs29941	19	*KCTD15*	G	−	0.56	−	0.88	−	1.00	+	0.77
	rs13078807	3	*CADM2*	G	−	0.19	−	0.42	+	0.92	−	0.41
	rs10767664	11	*BDNF*	A	+	0.25	+	0.66	−	0.99	−	0.80
	rs4771122	13	*MTIF3*	G	+	0.61	+	0.99	−	0.92	+	1.00
Women	rs9816226	3	*ETV5*	T	−	0.08	−	0.96	+	0.08	−	0.04
	rs2287019	19	*GIPR*	C	−	0.04	+	0.12	+	0.003	−	0.18
	rs1558902	16	*FTO*	A	−	0.55	+	0.009	+	0.001	+	0.55
	rs11847697	14	*PRKD1*	T	+	0.38	−	0.94	−	0.52	−	0.87
	rs7359397	16	*SH2B1*	T	−	0.15	−	0.47	+	0.30	−	0.23
	rs2241423	15	*MAP2K5*	G	−	0.31	−	0.27	−	0.50	−	0.90
	rs7138803	12	*FAIM2*	A	−	0.76	+	0.18	+	0.39	+	0.30
	rs10150332	14	*NRXN3*	C	−	0.90	+	0.31	+	0.10	−	0.64
	rs12444979	16	*GPRC5B*	C	−	0.40	−	0.71	+	0.76	−	0.07
	rs887912	2	*FANCL*	T	−	0.11	−	0.83	+	0.17	−	0.13
	rs2112347	5	*FLJ35779*	T	+	0.14	+	0.002	+	0.01	+	0.32
	rs2815752	1	*NEGR1*	A	−	0.52	+	0.28	+	0.02	−	0.51
	rs4836133	5	*ZNF608*	A	−	0.72	+	0.61	+	0.14	−	0.32
	rs571312	18	*MC4R*	A	+	0.75	+	0.05	+	0.06	+	0.93
	rs3810291	19	*TMEM160*	A	+	0.63	+	0.91	−	0.52	+	0.76
	rs4929949	11	*RPL27A*	C	+	0.62	+	0.23	+	0.81	+	0.29
	rs713586	2	*RBJ, ADCY3*	C	−	0.05	−	0.86	+	0.23	−	0.06
	rs2890652	2	*LRP1B*	C	−	0.76	+	0.11	+	0.09	−	0.72
	rs987237	6	*TFAP2B*	G	−	0.37	−	0.95	+	0.32	−	0.37
	rs10968576	9	*LINGO2*	G	−	0.01	+	0.20	+	0.01	−	0.12
	rs13107325	4	*SLC39A8*	T	−	0.11	−	0.03	−	0.25	−	0.08
	rs3817334	11	*MTCH2*	T	+	0.90	+	0.69	+	0.81	+	0.97
	rs206936	6	*NUDT3*	G	−	0.54	+	0.90	+	0.47	−	0.38
	rs2867125	2	*TMEM18*	C	−	0.51	+	0.005	+	3.807E-05	−	0.43
	rs543874	1	*SEC16B*	G	−	0.38	−	0.91	+	0.43	−	0.28
	rs1555543	1	*PTBP2*	C	+	0.79	+	0.32	+	0.28	+	0.33
	rs1514175	1	*TNNI3K*	A	−	0.24	−	0.79	+	0.29	−	0.06
	rs10938397	4	*GNPDA2*	G	+	0.34	+	0.04	+	0.46	+	0.07
	rs29941	19	*KCTD15*	G	+	0.80	+	0.07	+	0.09	+	0.81
	rs13078807	3	*CADM2*	G	−	0.03	+	0.53	+	0.11	−	0.41
	rs10767664	11	*BDNF*	A	−	0.25	−	0.80	+	0.19	−	0.49
	rs4771122	13	*MTIF3*	G	+	0.19	+	0.32	+	0.40	+	0.82
Overall	rs9816226	3	*ETV5*	T	−	0.01	−	0.01	+	0.03	−	0.04
	rs2287019	19	*GIPR*	C	−	0.03	−	0.03	+	0.0001	−	0.14
	rs1558902	16	*FTO*	A	−	0.17	−	0.17	+	6.24E-07	+	0.67
	rs11847697	14	*PRKD1*	T	+	0.72	+	0.72	−	0.81	−	0.68
	rs7359397	16	*SH2B1*	T	−	0.63	−	0.63	+	0.03	+	0.94
	rs2241423	15	*MAP2K5*	G	−	0.20	−	0.20	−	0.66	−	0.18
	rs7138803	12	*FAIM2*	A	−	0.26	−	0.26	+	0.28	+	0.99
	rs10150332	14	*NRXN3*	C	+	0.74	+	0.74	+	0.005	+	0.50
	rs12444979	16	*GPRC5B*	C	−	0.19	−	0.19	+	0.21	−	0.11
	rs887912	2	*FANCL*	T	−	0.78	−	0.78	+	0.09	−	0.57
	rs2112347	5	*FLJ35779*	T	+	0.15	+	0.15	+	0.004	+	0.14
	rs2815752	1	*NEGR1*	A	−	0.03	−	0.03	+	1.921E-05	−	0.34
	rs4836133	5	*ZNF608*	A	−	0.23	−	0.23	+	0.04	−	0.11
	rs571312	18	*MC4R*	A	−	0.91	−	0.91	+	0.10	−	0.73
	rs3810291	19	*TMEM160*	A	−	0.64	−	0.64	+	0.49	−	0.99
	rs4929949	11	*RPL27A*	C	−	0.27	−	0.27	+	0.28	+	0.98
	rs713586	2	*RBJ, ADCY3*	C	−	0.09	−	0.09	+	0.45	−	0.24
	rs2890652	2	*LRP1B*	C	−	0.24	−	0.24	+	0.20	−	0.08
	rs987237	6	*TFAP2B*	G	−	0.09	−	0.09	+	0.09	−	0.18
	rs10968576	9	*LINGO2*	G	−	0.05	−	0.05	+	0.004	−	0.19
	rs13107325	4	*SLC39A8*	T	−	0.29	−	0.29	−	0.24	−	0.10
	rs3817334	11	*MTCH2*	T	+	0.82	+	0.82	+	0.42	+	0.89
	rs206936	6	*NUDT3*	G	−	0.68	−	0.68	+	0.63	−	0.19
	rs2867125	2	*TMEM18*	C	−	0.21	−	0.21	+	8.817E-05	−	0.26
	rs543874	1	*SEC16B*	G	−	0.25	−	0.25	+	0.10	−	0.48
	rs1555543	1	*PTBP2*	C	+	0.44	+	0.44	+	0.21	+	0.16
	rs1514175	1	*TNNI3K*	A	−	0.08	−	0.08	+	0.37	−	0.02
	rs10938397	4	*GNPDA2*	G	−	0.44	−	0.44	+	0.13	−	0.91
	rs29941	19	*KCTD15*	G	−	0.85	−	0.85	+	0.17	+	0.81
	rs13078807	3	*CADM2*	G	−	0.01	−	0.01	+	0.17	−	0.16
	rs10767664	11	*BDNF*	A	+	0.97	+	0.97	+	0.19	−	0.59
	rs4771122	13	*MTIF3*	G	+	0.45	+	0.45	+	0.45	−	0.87

All CT traits presented with the same coded allele, and all are modeled relative to the previously-published BMI trait-increasing allele. Z-statistic indicates direction relative to the coded allele.

### eQTL Results

In order to help identify the potential causal gene in the region surrounding our lead SNP, we first reviewed publically available databases, but did not identify any associations of rs1659258 with eQTLs (see methods for additional details). However, most of these databases consist of women and men in combined analyses; given that our GWAS finding was in women alone, we performed sex-specific eQTL analyses of rs1659258 in up to 848 patients (mean BMI 50.5 kg/m2) who underwent Roux-en-Y gastric bypass surgery who also underwent subcutaneous and visceral fat biopsy [40]. We performed eQTL testing in the 1 MB region surrounding rs1659258 (n = 31 genes). Using a corrected p-value threshold of p<0.05, only *THNSL2* expression in subcutaneous fat in women was associated with our lead SNP (p = 0.03) but not men (p = 0.96). We did not observe association with expression in VAT in either women or men.

## Discussion

### Principal Findings

We have uncovered a new locus for VAT at *THNSL2* in women that reveals a striking sexual dimorphism, where we observed significance only in women, but not men. We also observed genome-wide significance for rs11118316 at *LYPLAL1* for the VAT/SAT ratio; this region was previously identified in a GWAS for WHR in the GIANT consortium, although our lead SNP is only in moderate LD with the SNP identified by GIANT. Finally, we performed targeted SNP evaluations of 14 SNPs previously identified in association with central fat distribution and identified nominal associations at 7 loci.

### In the Context of the Current Literature

Prior genome-wide association studies have focused on using anthropometric measures to define body fat distribution. The GIANT consortium identified 14 loci associated with WHR adjusted for BMI [32], the majority of which demonstrated stronger associations in women as compared to men. More recently, a SNP at the *IRS1* locus was identified in association with body percent fat in men, but not women [41]. We showed that the VAT/SAT ratio in men was associated with this same *IRS1* SNP [41]. Taken together, these prior data suggest that additional loci may exist in association with fat distribution above and beyond those associated with generalized adiposity. In the present study, we identified a new locus in association with VAT in women, highlighting the utility of more precise phenotyping of abdominal fat distribution.

We also extend the observations of a marked sexual dimorphism that have been made using primarily anthropometric measurements to CT-imaged fat depots. It is well-known that in women as compared with men, VAT levels are relatively lower, and SAT levels are higher [42]. This gender difference underscores the need to consider women and men separately in assessing the genetic architecture of fat distribution. We also note that our most significant SNP at the *LYPLAL1* locus was associated with the VAT/SAT ratio in both women and men as compared to only WHR in GIANT [32], while the lead SNP identified in GIANT demonstrated some evidence for heterogeneity by sex with the VAT/SAT ratio. Taken together, these findings highlight the potential differences in directly-imaged fat distribution traits as compared to anthropometric data.

An important consideration is whether our finding of rs1659258 at *THNSL2* in association with VAT in women represents a variant that is specific to central adiposity. Indeed, our strongest result is derived from VAT itself. However, we observed associations with SAT in women, and our validation data suggests this SNP is also associated with BMI in women. Once we adjusted the VAT trait for BMI, our SNP did not completely lose its statistical significance (p = 6.9*10E-05), suggesting that there is some specificity of our finding to fat deposition in the central abdominal compartment. This result is in contrast to our prior results for a SNP in *NRXN3* in association with waist circumference: upon adjustment for BMI, statistical significance was attenuated [43]. Finally, our eQTL data reveals nominal expression in subcutaneous but not visceral adipose tissue.

### Potential Underlying Biology

Our lead SNP for VAT in women, rs1659258, is located in an intergenic region upstream from *THNSL2* and *FABP1*. However, rs1659258 is not in linkage disequilibrium (LD) with any coding SNPs within 88,200-88,700 kb. In addition, the correlations between rs1659258 with coding SNPs in *FOXI3*, *C2orf51*, *THNSL2*, *EIF2AK3*, *FABP1*, and *SMYD1* genes are low (r2<0.15). Finally, there is no evidence that 2p11-p12, where rs1659258 is located, has been previously implicated in association with copy number variation in adipose-related human disease. Nonetheless, we explored the potential biology in this region. Fatty acid binding protein is produced in the liver and is involved with fatty acid metabolism. Free fatty acid flux has previously been shown to be more strongly associated with visceral as compared to subcutaneous fat [44]. In addition, women have been shown to have a faster rate of non-oxidative free fatty acid disposal as compared to men, but without concomitant worsened metabolic risk factor profiles [45]. While *FABP1* represents an exciting potential candidate gene, rs1659258 resides in a neighboring linkage disequilibrium block that does not contain any genes. *THNSL2* is just downstream of *FABP1* and our lead SNP demonstrates nominal gene expression to *THNSL2*, which is part of the threonine synthase family. A recent analysis of RNA expression in 225 healthy Pima Indian skeletal muscle biopsies showed a bimodal (ie two discrete clusters) expression of *THNSL2*, thought to occur due to cis-acting polymorphisms [46].

It is particularly notable that we uncovered a genome-wide significant finding with rs11118316 at *LYPLAL1* for the VAT/SAT ratio overall. The VAT/SAT ratio is a metric of propensity to store visceral as compared to subcutaneous fat and has been shown to be associated with cardiometabolic risk [47]. We previously observed associations of a SNP in this gene in association with WHR-adjusted-for-BMI in the GIANT consortium [32], and *LYPLAL1* has also been associated with cardiometabolic traits [48] and fatty liver [49]. *LYPLAL1* encodes the lysophospholipase-like protein 1 and has been shown to be upregulated in the visceral and subcutaneous fat of obese subjects [50].

There are several potential explanations that could potentially account for our observed genetic association, particularly the marked sexual dimorphism. It has been previously shown that the familial contribution to fat distribution phenotypes is stronger in women as compared to men [33]. This concept is further strengthened by findings in mice suggesting that gonadal hormones are important in the sex-specific expression of genes related to metabolic traits [51]. In addition, known gender differences in fat distribution may also in part contribute to our findings, as women have been shown to have more subcutaneous fat but less visceral fat compared to men [20]. The relatively smaller amount of visceral fat in women may have increased our ability to detect a genetic signal. Finally, we have consistently observed stronger associations among women as compared to men with respect to metabolic risk factors in association with VAT [20]. While the reasons for this have not been fully elucidated, the stronger associations in women as compared to men is similar to what we have observed in the present analysis and in a prior GWAS of fat distribution phenotypes [32].

### Strengths and Limitations

Strengths of our study include directly measured visceral and subcutaneous fat using CT imaging. Phenotyping using imaging is superior to typical anthropometric measures in the ability to partition the subcutaneous from visceral fat depots. Limitations include sample size: because of the limited number of studies with these imaging measurements and genome-wide association data, our discovery sample size was modest compared with other contemporary analyses. However, we note that performing sex-specific analyses actually enabled us to uncover a new locus, highlighting how heterogeneity can mask findings even when sample sizes are larger. Finally, the mean BMI in our gastric bypass eQTL dataset was substantially higher than the mean BMI in our discovery GWAS, which may affect generalizability of the eQTL data.

### Conclusions

We have uncovered new loci for body fat distribution phenotypes, highlighting that loci exist for fat distribution that are largely independent of overall adiposity. More refined phenotypes for body composition and fat distribution can detect new loci not uncovered in large-scale GWAS of anthropometric traits.

## Methods

### Phenotype Definition

VAT and SAT were measured on CT following protocols established by each study as detailed in the Study-Specific Methods. We created sex-specific residuals adjusting for age, age-squared, smoking and principal components derived from genotypes denoting population stratification. The following traits were created in the overall sample and in women and men separately for each participating study: VAT, SAT, VAT-adjusted-for-BMI, and VAT/SAT ratio. VAT-adjusted-for-BMI provides insight into the relative amount of VAT controlling for the degree of generalized adiposity, whereas the VAT/SAT ratio is a metric of the propensity to deposit fat viscerally as compared to subcutaneously. The VAT/SAT ratio has been previously shown to be associated with cardiometabolic risk factors [47]. The correlation between VAT-adjusted-for-BMI and the VAT/SAT ratio in the Family Heart Study is 0.76 (p<0.0001; N = 2658).

### Heritability Analyses

We created sex-and-cohort specific residuals, which were then pooled and analyzed using variance components analysis (SOLAR) [52].

### Genotyping

Genotyping was conducted as specified in the Table S1 and the Study Specific Methods. We applied quality-control filters in order to exclude low-quality SNPs or samples. Based on CEU samples, each study imputed ∼2.5 million Phase 2 HapMap SNPs. We used imputed allelic dosage in the analysis. Additional details can be found in Table S1.

### Primary Association Analyses and Meta-Analysis

The primary analysis was conducted in each cohort separately using regression analysis, assuming additive genetic effects and accounting for dependence among family member when appropriate. We accounted for principal components where necessary in order to prevent population stratification as well as the assumption of homogeneity within samples of European ancestry. These results were gathered together and used to conduct a fixed effects weighted Z meta-analysis (to allow for differences in phenotype scaling across the participating studies) using METAL [53]. We have previously shown that the association between single-slice VAT and SAT measurements at the L4/L5 level is highly correlated with volumetric measurements (r = 0.95–0.99) [54]. This approach weights the signed Z statistics from each study by its sample size to obtain a weighted sum, from which a p value is obtained. We applied the genomic control correction to control type I error rates. SNPs that reached a meta-analysis *P* value≤5×10^−8^ were considered to be genome-wide significant [55].

### Validation Analysis

Stage 2 validation was conducted for our novel lead SNP, rs1659258, using data from studies not part of the present meta-analysis in the GIANT consortium data in sex-specific meta-analyses for BMI and waist circumference (personal communication, Heid I et al). P-values for SNPs discovered in the present effort in association with adiposity phenotypes reported by the GIANT consortium were queried. We concluded significant results when a direction-consistent p-value was at least p<0.05.

### Analyses of Related Phenotypes

For each validated SNP, we obtained sex-specific association results for lipid and glycemic phenotypes from the MAGIC and GLGC consortia.

### Interaction Testing

We performed formal tests of interaction of rs1659258 by sex (women as compared to men) and age (stratified above/below age 60). Briefly, each study generated the interaction regression coefficient, its standard error and its p value through regression. For the sex interaction, we included, age, age-squared, smoking (yes/no), sex, and any principal components (and study center) that were used in the original discovery analysis. We additionally added rs1659258 and the cross-product rs1659258*sex. The age interaction analysis was performed in women only. Because the AGES and Health ABC studies only included participants older than 65 years, they did not contribute to this analysis. Model covariates included age, age-squared, smoking (yes/no), and any principal components (and study center) with the addition of rs1659258 and the cross-product of rs1659258 and the dichotomized age term. We meta-analyzed the interaction terms across all studies using the weighted z-score approach.

### eSNP Analysis

Using publically available datasets, we tested for the association of rs1659258 in expression SNPs (eSNP) datasets comprised of the following tissue types: lymphocytes [56], leukocytes [57], leukocytes from patients with Celiac disease [58], lymphoblastoid cell lines (LCL) from children with asthma [59], HapMap LCL from 3 populations [60], a separate study on HapMap CEU LCL [61], peripheral blood monocytes [62], [63], adipose tissue [40], [64], and blood samples [64], 2 studies on brain cortex [62], [65], three large studies of brain regions including prefrontal cortex, visual cortex and cerebellum (Emilsson, personal communication), liver [40], [66], osteoblasts [67], skin [68], and additional fibroblast, T cell and LCL samples [69]. We considered significance to be the association with gene transcript levels as originally described.

For sex-specific expression data, we queried data from patients who underwent a Roux-en-Y gastric bypass [40]. Briefly, we queried a 1 MB region surrounding our lead SNP for VAT in women and men separately. Altogether, 31 genes were included in the query. Statistical significance was considered to be a p-value<0.05 after correction for multiple testing.

### Study-Specific Information: Framingham Heart Study

In 1948, the Framingham Heart Study began when the Original Cohort was enrolled [70]. Beginning in 1971, the Offspring Cohort was enrolled (5,124 participants); the methodology and design has been described. In 2002, the Third Generation cohort was enrolled (n = 4095) [71]. Participants for this study were drawn from the Framingham Heart Study Multi-detector Computed Tomography (MDCT) Study, a population-based sub-study of the community-based Framingham Heart Study Offspring and Third Generation cohorts. Participants for the current study were drawn from the MDCT sub-study. Between June 2002 to April 2005, 3529 participants (2111 Third Generation, 1418 Offspring participants) underwent MDCT assessment of coronary and aortic calcium. Inclusion in this study was weighted towards participants from larger Framingham Heart Study families and those who resided in the Greater New England area. Men had to be at least 35 years of age, women had to be at least 40 years of age and non-pregnant, and all participants had to weigh less than 350 pounds. Of the total of 3529 subjects imaged, 3394 had interpretable CT measures, 3329 of whom had both SAT and VAT measured, and 3158 participated in the present GWAS study.

We observed association with the first principal component estimated using Eigenstrat [72]; this component was included in our regression models.

#### Volumetric adipose tissue imaging

Subjects underwent eight-slice MDCT imaging of the chest and abdomen in a supine position as previously described (LightSpeed Ultra, General Electric, Milwaukee, WI) [73]. Briefly, twenty-five contiguous five mm thick slices (120 kVp, 400 mA, gantry rotation time 500 ms, table feed 3∶1) were acquired covering 125 mm above the level of S1.

#### Abdominal adipose tissue measurements

Subcutaneous and visceral adipose tissue volumes (SAT and VAT) were assessed (Aquarius 3D Workstation, TeraRecon Inc., San Mateo, CA). In order to identify pixels containing fat, an image display window width of −195 to −45 Hounsfield Units (HU) and a window center of −120 HU were used. The abdominal muscular wall separating the visceral from the subcutaneous compartment was manually traced. Inter-reader reproducibility was assessed by two independent readers measuring VAT and SAT on a subset of 100 randomly selected participants [73]. Inter-class correlations for inter-reader comparisons were 0.992 for VAT and 0.997 for SAT. Similar high correlations were noted for intra-reader comparisons.

#### Imputation

As a reference panel for imputation, we used Phase II CEU HapMap individuals; we imputed genotypes to nearly 2.5 million HapMap SNPs; further details are presented in Table S1. We used MACH v1.0.15/16 (http://www.sph.umich.edu/csg/abecasis/MACH/) in conjunction with 200 (101 Men and 99 Women) biologically independent individuals to establish parameter estimates and then used the parameter estimates to infer gene dosage for all study participants. We expressed imputed genotypes as allelic dosage (which is a decimal value ranging from 0–2).

#### Statistical analysis

We performed linear mixed effects regression modeling for SAT, VAT, and the VAT/SAT ratio to account for pedigree structure (using the R lme and kinship package).

### Study-Specific Information: Family Heart Study

The Family Heart Study (FamHS) is a multicenter, population-based, family study designed to investigate the determinants of cardiovascular disease [74]. Families in the FamHS were selected at random (588 families) or ascertained for family history of CHD (656 families) using information collected in the parent studies—Framingham Heart Study (Framingham, MA, USA), the Utah Health Family Tree Study (Salt Lake City, UT, USA) or the Atherosclerosis Risk in Communities Study (Minneapolis Suburbs, MN, USA and Forsyth County, NC,USA). Between 2002 and 2003 about two-thirds of the largest families were invited to participate in a follow-up clinical examination that included measurement of the liver and abdomen with cardiac CT using standardized procedures and quality control methods developed in NHLBI's MESA and CARDIA studies [75]. Informed consent was obtained from all participants, and this project was approved by the Institutional Review Boards of all participating institutions.

#### CT scan–related phenotypes

Participants underwent a cardiac MDCT exam with four detectors using a standardized protocol as described previously [75]. For participants weighing 100 kg (220 lbs) or greater, the mAs were increased by 25%. The effective radiation exposure for the average participant of each coronary scan was 1.5 mSv for men and 1.9 mSv for women. Participants received two sequential scans. CT images from all study centers were sent electronically to the central CT reading center located at Wake Forest University Health Sciences, Winston Salem, NC, USA.

CT scans of the abdomen were reconstructed into 5 mm slices with the maximum 50 cm field-of-view to include the whole abdomen for body composition. Total and adipose tissues were measured volumetrically from two 5 mm contiguous slices located at the level of the lumbar disk between the 4^th^ and 5^th^ vertebra so as to be comparable to a single 10 mm slice used historically. Tissues with an attenuation between −190 to −30 Hounsfield units was define as adipose tissue. The MIPAV application (http://mipav.cit.nih.gov/index.php) was used by experienced analysts to segment the images based on anatomic boundaries (skin, subcutaneous fat-muscle interface and peritoneum) into the entire abdomen, abdominal wall and intra-abdominal compartments [76]. In each compartment the total volume and fat volumes were determined allowing calculation of the total abdominal adipose tissue, subcutaneous adipose tissue and visceral adipose tissue contained with the 10 mm slice located at L4–5.

#### Statistical analysis

We performed linear mixed effects regression modeling for SAT, VAT, and the VAT/SAT ratio, accounting for dependency among family member as a function of their kinship correlation (R kinship package).

### Study-Specific Information: HABC Study

The Health ABC study is a prospective cohort study investigating the associations between body composition, weight-related health conditions, and incident functional limitation in older adults. Health ABC enrolled well-functioning, community-dwelling black (n = 1281) and white (n = 1794) men and women aged 70–79 years between April 1997 and June 1998. Participants were recruited from a random sample of white and all black Medicare eligible residents in the Pittsburgh, PA, and Memphis, TN, metropolitan areas. Participants have undergone annual exams and semi-annual phone interviews. The current study sample consists of 1559 white participants who attended the second exam in 1998–1999 with available genotyping and SAT/VAT data.

Regional fat depots were assessed from CT scans obtained in Pittsburgh on a General Electric 9800 Advantage (General Electric, Milwaukee, WI) and in Memphis on a Siemens Somatron Plus 4 (Siemens, Erlangen, Germany) or Picker PQ2000S (Marconi Medical Systems, Cleveland, OH). A single axial scan (140 kVp, 300 to 360 mAs, 10-mm thickness) was taken at the disk space between the fourth and fifth lumbar vertebrae. Images were transferred to the Reading Center at the University of Colorado Health Sciences Center on optical disc or magnetic tape. Analyses were performed on a SPARC station II (Sun Microsystems, Mountain View, CA) using IDL development software (RSI Systems, Boulder, CO). An outline was traced surrounding the abdominal cavity. The adipose tissue density range was determined with a bimodal image distribution histogram for each participant. Visceral fat was defined as the area of all adipose tissue within the abdominal cavity with exclusion of the muscle region, calculated by multiplying the number of pixels within this range by a single pixel area. Abdominal subcutaneous fat was defined as the difference in the area between the entire adipose tissue in the scan and visceral fat. To assess the reproducibility of these measurements, 5% of the data was re-read in a blinded fashion. The intra-class correlation coefficients of reliability ranged from 0.93 to 1.000.

#### Genotyping and imputation

Genomic DNA was extracted from buffy coat collected using PUREGENE DNA Purification Kit during the baseline exam. Genotyping was performed by the Center for Inherited Disease Research (CIDR) using the Illumina Human1M-Duo BeadChip system. Samples were excluded from the dataset for the reasons of sample failure, genotypic sex mismatch, and first-degree relative of an included individual based on genotype data. Genotyping was successful for 1,151,215 SNPs in 2,802 unrelated individuals (1663 Caucasians and 1139 African Americans). Imputation was done for the autosomes using the MACH software version 1.0.16. SNPs with minor allele frequency ≥1%, call rate ≥97% and HWE p≥10-6 were used for imputation. HapMap II phased haplotypes were used as reference panels. For EAs, genotypes were available on 914,263 high quality SNPs for imputation based on the HapMap CEPH reference panel (release 22, build 36). A total of 2,543,887 in EAs are available for analysis.

#### Statistical analysis

We performed linear regression modeling for SAT, VAT, and the VAT/SAT ratio. We observed association with the first principal components estimated using Eigenstrat [72]; this was accounted for in our analyses.

### Study-Specific Information: Age, Gene/Environment Susceptibility–Reykjavik Study

The AGES-Reykjavik study is an ongoing study of the effects of gene-environment interactions and other risk factors on disease in old age. AGES-Reykjavik is a subset of a larger population based cohort study called the Reykjavik-study. The aim of the original study was to prospectively investigate risk factors for cardiovascular disease in the Icelandic population. The original Reykjavik-cohort was established in 1967 with a random sample of 30,795 individuals born in the years 1907–1935, and residing in Reykjavik, the capital of Iceland. A total of 18,045 individuals entered the study as participants and attended examinations. The AGES-Reykjavik sample was constructed in 2002 by randomly drawing 8,030 individuals who were still alive from the original Reykjavik-cohort (*n* = 11,459). A total of 5,764 individuals (58% women) entered the AGES-Reykjavik study as participants. All cohort members were European Caucasians.

The AGES-Reykjavik Study was designed to investigate aging using a multifaceted comprehensive approach. Physical, physiological and questionnaire examinations were conducted in three visits for each subject including detailed medical history, physical examination, laboratory and screening tests, and questionnaires on health-related behaviors such as alcohol consumption, smoking history, and physical activity. Pertinent to this study, these measures included anthropometric measurements and computerized tomography measures of adipose depots in the abdomen and thigh. The AGES–Reykjavik study was approved by the institutional review boards of the National Institute on Aging and the Icelandic National Bioethics Committee (VSN: 00-063), and written informed consent was obtained from all participants.

#### Analytic sample

Of the 5,764 individuals who agreed to participate in the AGES-Reykjavik study, 5,427 individuals attended the research center for examinations while 337 received a home visit. A total of 204 individuals did not contribute CT-data at the research center. However, among those individuals with CT, only 3,664 had genotyping at the Laboratory of Neurogenetics, Intramural Research Program, NIA, Bethesda, Maryland, and 3,219 participants passed QC criteria for genotyping. Of these, 3172 had complete genotyping and complete CT data for abdominal subcutaneous and visceral adipose depots.

#### Image acquisition

Images for abdominal adipose depots were acquired in a single 10 mm thick trans-axial section of the abdomen at the level of the L4–L5 vertebrae using a Siemens Somatom Sensation 4 multi-detector CT scanner (Siemens Medical Solutions, Erlangen, Germany) (standard scan setting: slice thickness: 10 mm, tube voltage; 140 kilo-voltage, tube-current-time-product; 50 milli-ampere-seconds and scan time 0.361 sec). Study participants weighing more than 110 kg (kilo-grams) underwent CT with a tube current setting that was 25% higher than the standard scan setting. The images were reconstructed into a display field of view of 350 mm to include a calibration phantom (Image Analysis, Columbia, KY, USA) which was positioned under the abdomen of each subject. VAT area was estimated from all pixels in the abdominal cavity within the range of −50 to −200 Hounsfield units. Inter-observer variability based on the re-analysis of randomly selected 365 scans from the core study population by an expert observer showed an average correlation coefficient of 0.99. Intra-observer variability based on re-analysis of 45 scans by each of the four observers resulted in an average correlation coefficient of 0.99.

#### Imputation

As a reference panel for imputation, we used Phase II CEU HapMap individuals; we imputed genotypes to nearly 2.5 million HapMap SNPs; further details are presented in Table S1.

#### Statistical analysis

We performed linear regression modeling for SAT, VAT, and the VAT/SAT ratio.

## Supporting Information

Figure S1 Q-q plots for all traits. VATSAT is the VAT/SAT ratio, and VATaBMI is VAT-adjusted-for-BMI.
(TIF)

Figure S2 Manhattan plots for all traits. VATSAT is the VAT/SAT ratio, and VATaBMI is VAT-adjusted-for-BMI.
(PPT)

Table S1 Genotyping information from each study.
(DOC)

Table S2 All results for All Traits with P-values<9.9*10E-06; all SNPs are presented with the coded allele in the effect raising direction. Unique SNPs at each loci within each meta-analysis were identified and then subsequently pooled. Sample sizes are as follows: SAT (overall: 10557, women 5560, men 4995); VAT (overall 10557, women 5560, men 4997); VATSAT ratio (overall 10556, women 5559, men 4997), VAT-adj-BMI (overall 10542, women 5549, men 4993). VATSAT is the VAT/SAT ratio, and VATaBMI is VAT-adjusted-for-BMI.
(DOC)

Text S1 Investigator names for the MAGIC Consortium.
(DOC)
