# Supplementary figures and images for: Genome-Wide Association for Abdominal Subcutaneous and Visceral Adipose Reveals a Novel Locus for Visceral Fat in Women
Source: PLoS Genet. 2012 May 10;8(5):e1002695. doi: 10.1371/journal.pgen.1002695 (PMC3349734; doi:10.1371/journal.pgen.1002695)

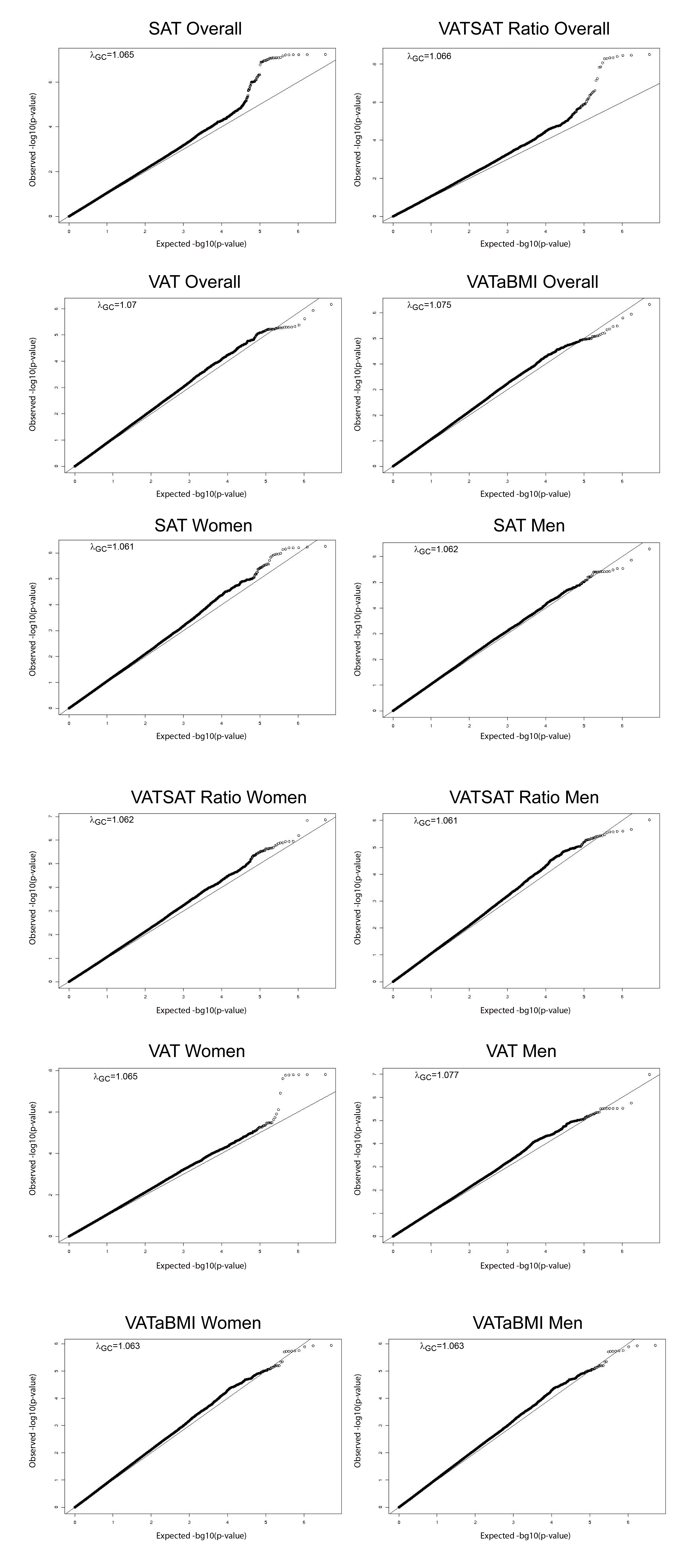

Supplement: Figure S1 — Q-q plots for all traits. VATSAT is the VAT/SAT ratio, and VATaBMI is VAT-adjusted-for-BMI. (TIF) [file pgen.1002695.s001.tif]
