# Supplementary material for: Genome-Wide Association for Abdominal Subcutaneous and Visceral Adipose Reveals a Novel Locus for Visceral Fat in Women
Source: PLoS Genet. 2012 May 10;8(5):e1002695. doi: 10.1371/journal.pgen.1002695 (PMC3349734; doi:10.1371/journal.pgen.1002695)
Supplement: Figure S2 — Manhattan plots for all traits. VATSAT is the VAT/SAT ratio, and VATaBMI is VAT-adjusted-for-BMI. (PPT) [file pgen.1002695.s002.ppt]

## Slide 1
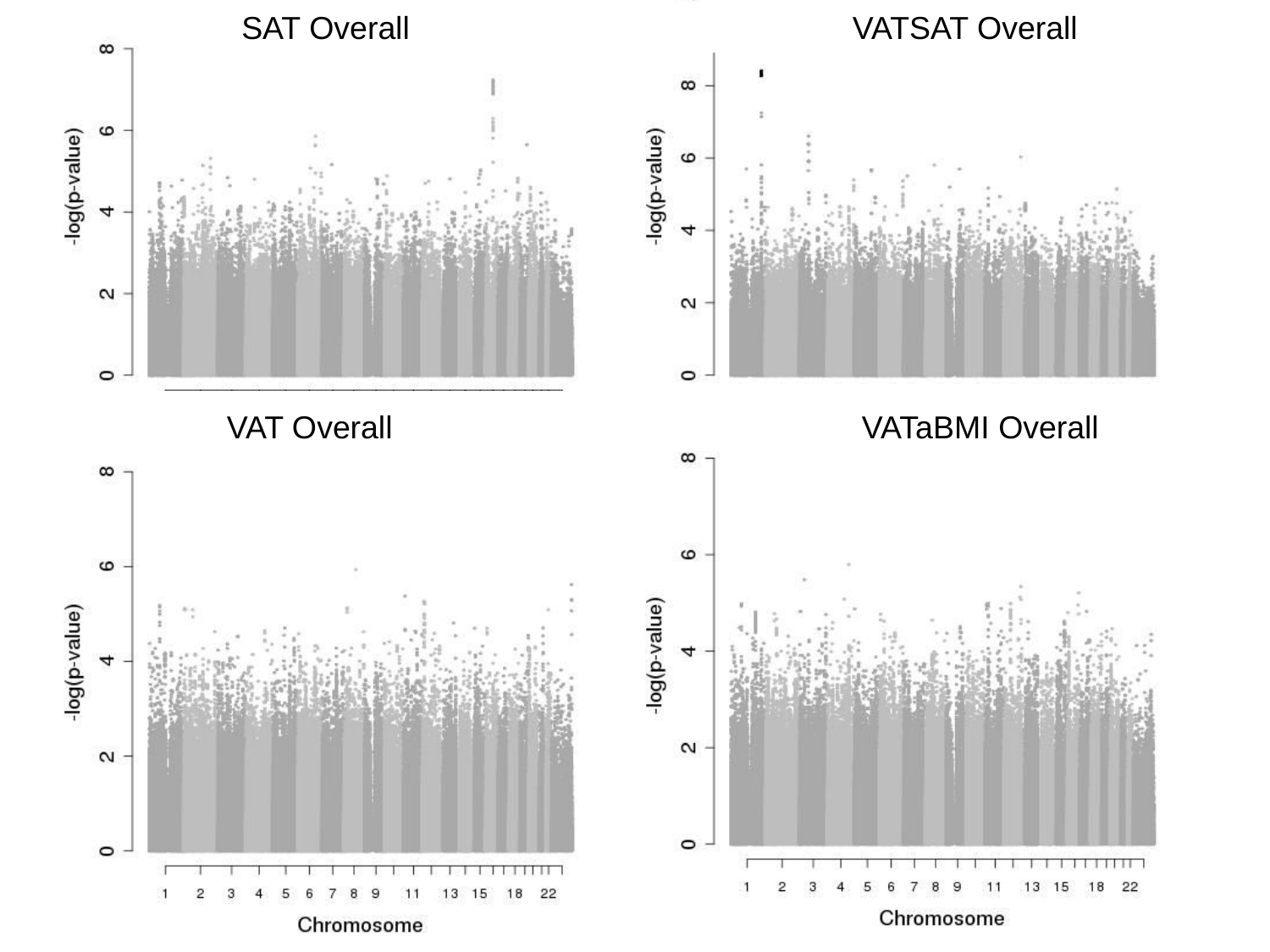

SAT Overall VATSAT Overall
VAT Overall VATaBMI Overall

## Slide 2
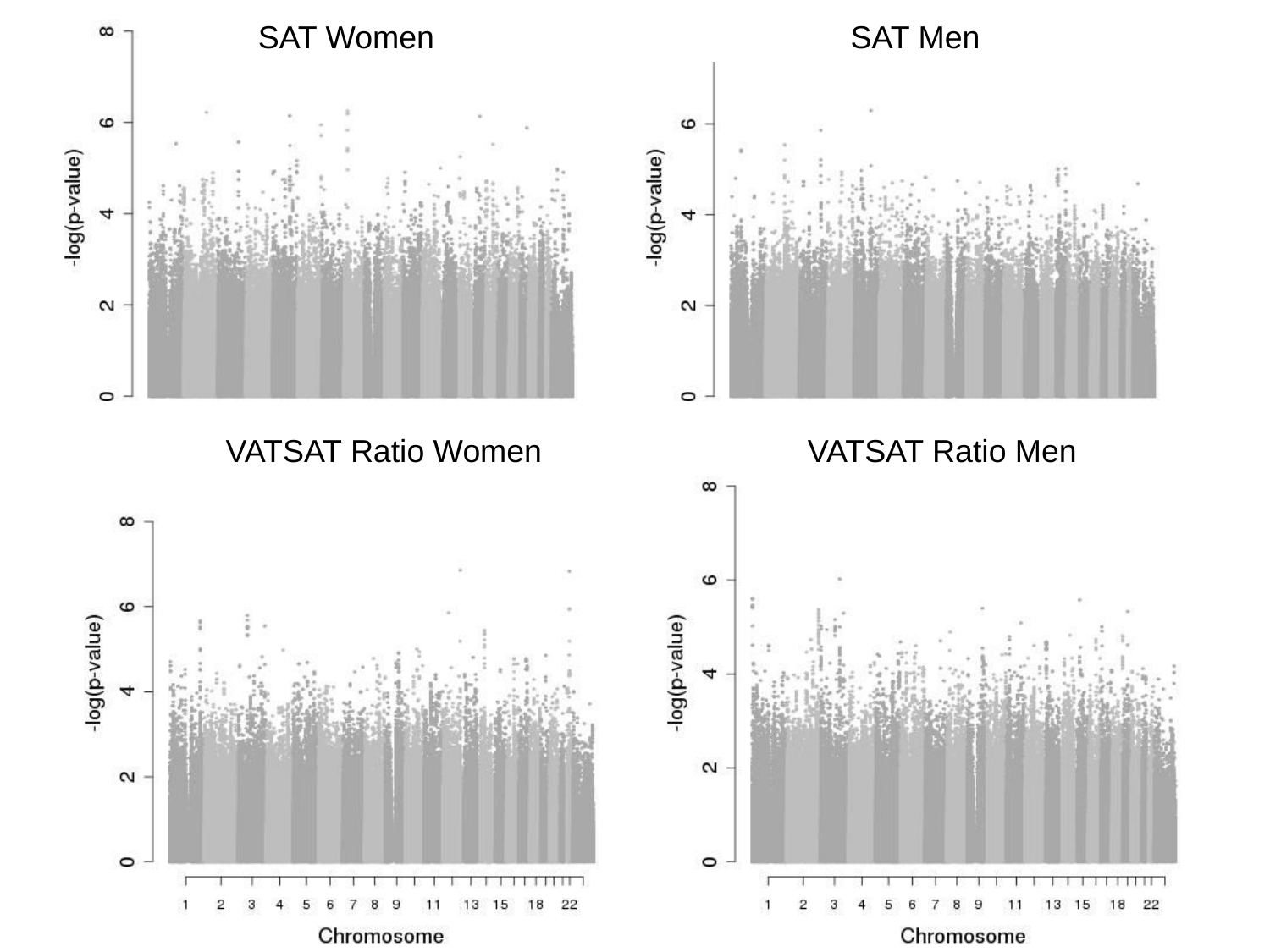

SAT Women SAT Men
 VATSAT Ratio Women VATSAT Ratio Men

## Slide 3
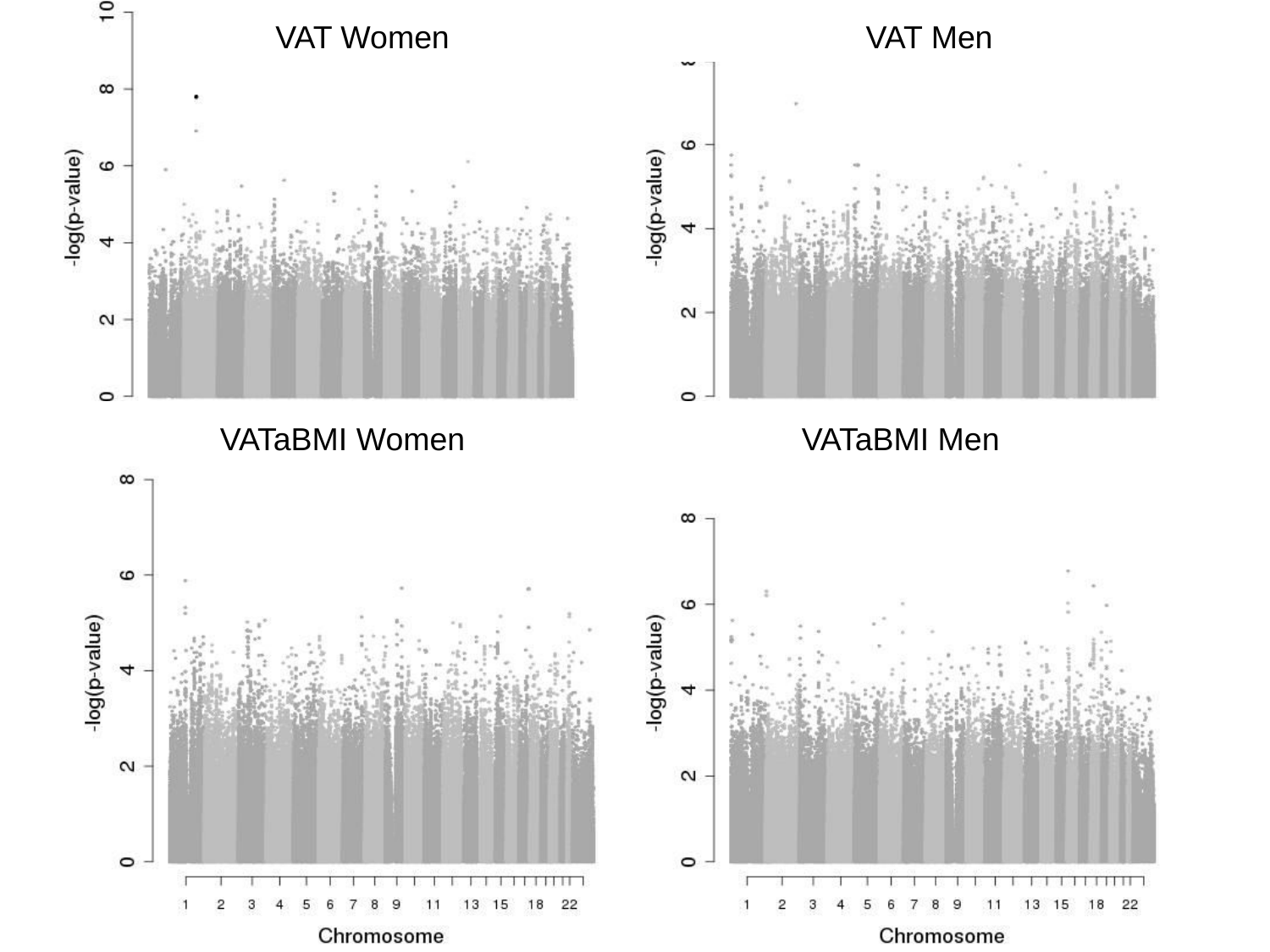

VAT Women VAT Men
VATaBMI Women VATaBMI Men
