# Supplementary material for: Genome-Wide Association for Abdominal Subcutaneous and Visceral Adipose Reveals a Novel Locus for Visceral Fat in Women
Source: PLoS Genet. 2012 May 10;8(5):e1002695. doi: 10.1371/journal.pgen.1002695 (PMC3349734; doi:10.1371/journal.pgen.1002695)
Supplement: Table S1 — Genotyping information from each study. (DOC) [file pgen.1002695.s003.doc]

| Study | Array type | Genotype calling | QC filters for genotyped SNPs used for imputation | No of SNPs used for imputation | Imputation | Imputation Backbone for phased CEU haplotypes (NCBI build) | Filtering of imputed genotypes | Data management and statistical analysis |
| --- | --- | --- | --- | --- | --- | --- | --- | --- |
| Framingham Heart Study | Affymetrix 500K  Affymetrix 50K supplemental | Affymetrix | pHWE<1e-6, call rate<97%, mishap p<1e-9, MAF<0.01, Mendelian errors>100, SNPs not in Hapmap or strandedness issues merging with Hapmap | 378,163 | MACH version 1.0.15 | HapMap release 22 (build 36) | none | R, linear mixed effect models and GEE models, robust variance option to account for relatedness |
| Health ABC | Illumina 1M | BeadStudio v3.3.7 | minor allele frequency ≥ 1%, call rate ≥97% and HWE p≥10^-6^ | 914,263 | MACH v1.0.16 | HapMap CEPH release 22 (build 36) | None | R, linear regression and logistic regression models |
| Family Heart Study | Illumina550K | Illumina | MAF < 1%, pHWE<10E-6 | 456,293 | MACH version 1.0.15 | phased CEU haplotypes, HapMap release 22 (build 26) | none | SAS |
| AGES | Illumina Hu370CNV | Illumina | pHWE<1e-6, call rate<97%, mishap* p<1e-9, MAF<0.01, , SNPs not in Hapmap or strandedness issues merging with Hapmap | 329804 | MACH version 1.0.16 | HapMap release 22 (build 36) | none | R,ProbABEL, Linear and Logistic Regression |

* Mishap refers to non random missingness by haplotype
