# Supplementary material for: Genome-Wide Association for Abdominal Subcutaneous and Visceral Adipose Reveals a Novel Locus for Visceral Fat in Women
Source: PLoS Genet. 2012 May 10;8(5):e1002695. doi: 10.1371/journal.pgen.1002695 (PMC3349734; doi:10.1371/journal.pgen.1002695)
Supplement: Table S2 — All results for All Traits with P-values<9.9*10E-06; all SNPs are presented with the coded allele in the effect raising direction. Unique SNPs at each loci within each meta-analysis were identified and then subsequently pooled. Sample sizes are as follows: SAT (overall: 10557, women 5560, men 4995); VAT (overall 10557, women 5560, men 4997); VATSAT ratio (overall 10556, women 5559, men 4997), VAT-adj-BMI (overall 10542, women 5549, men 4993). VATSAT is the VAT/SAT ratio, and VATaBMI is VAT-adjusted-for-BMI. (DOC) [file pgen.1002695.s004.doc]

| Trait | rsnumber | Chr | Physical Location | Coded Allele | Other Allele | Coded Allele Frequency | P.value | Nearest Gene |
| --- | --- | --- | --- | --- | --- | --- | --- | --- |
| VATSAT overall | rs11118316 | 1 | 217723786 | a | g | 0.44 | 3.13E-09 | *LYPLAL1* |
| VAT women | rs1659258 | 2 | 88440703 | a | g | 0.92 | 1.58E-08 | *THNSL2* |
| SAT overall | rs9922619 | 16 | 52389272 | t | g | 0.44 | 5.87E-08 | *FTO* |
| VAT men | rs2241193 | 2 | 217262458 | c | t | 0.89 | 1.04E-07 | *IGFBP5* |
| VATSAT women | rs1316952 | 12 | 122965503 | t | c | 0.87 | 1.38E-07 | *DNAH10* |
| VATaBMI men | rs11643447 | 16 | 6639262 | t | a | 0.05 | 1.67E-07 | *A2BP1* |
| VATSAT overall | rs7433808 | 3 | 64702126 | a | t | 0.74 | 2.49E-07 | *ADAMTS9* |
| VATaBMI men | rs1791780 | 18 | 22146670 | g | a | 0.54 | 3.72E-07 | *TAF4B* |
| VATaBMI overall | rs1641895 | 16 | 12548616 | t | c | 0.02 | 4.73E-07 | *LOC92017* |
| VATaBMI men | rs10495537 | 2 | 6611970 | t | c | 0.32 | 4.94E-07 | *LOC129607* |
| SAT men | rs13166814 | 5 | 117365652 | a | g | 0.16 | 5.05E-07 | *DTWD2* |
| SAT women | rs7833268 | 8 | 23874632 | c | g | 0.13 | 5.58E-07 | *STC1* |
| SAT women | rs1722636 | 2 | 161490019 | t | c | 0.23 | 5.98E-07 | *TANK* |
| VATSAT overall | rs12204127 | 6 | 90754995 | t | c | 0.20 | 6.06E-07 | *BACH2* |
| VATSAT women | rs743793 | 22 | 31891746 | t | c | 0.54 | 6.31E-07 | *LARGE* |
| VAT overall | rs16909318 | 8 | 82607779 | c | a | 0.88 | 6.89E-07 | *LOC646486;FABP4* |
| SAT women | rs17501712 | 5 | 123599059 | t | g | 0.14 | 7.13E-07 | *ZNF608* |
| SAT women | rs12591650 | 15 | 58847999 | a | g | 0.85 | 7.29E-07 | *RORA* |
| VAT women | rs3783938 | 14 | 80664133 | t | c | 0.07 | 7.72E-07 | *TSHR* |
| VATSAT overall | rs1316952 | 12 | 122965503 | t | c | 0.87 | 9.44E-07 | *DNAH10* |
| VATSAT men | rs11712655 | 3 | 136707939 | t | g | 0.72 | 9.50E-07 | *EPHB1* |
| VATaBMI men | rs8106493 | 19 | 38405768 | t | c | 0.20 | 1.06E-06 | *SLC7A10* |
| SAT women | rs11725509 | 4 | 149531178 | a | c | 0.97 | 1.11E-06 | *NR3C2* |
| VATaBMI overall | rs11930273 | 4 | 151121207 | g | a | 0.91 | 1.14E-06 | *DCLK2* |
| VATaBMI women | rs2798334 | 1 | 112238867 | c | t | 0.73 | 1.16E-06 | *KCND3* |
| VATaBMI women | rs7956193 | 12 | 111976167 | t | c | 0.29 | 1.19E-06 | *DTX1;OAS2;RASAL1* |
| VATSAT women | rs1567127 | 11 | 132335072 | g | a | 0.20 | 1.30E-06 | *OPCML* |
| SAT women | rs7245708 | 19 | 50887596 | c | a | 0.04 | 1.31E-06 | *QPCTL;FBXO46;EML2;SNRPD2;GIPR* |
| VATaBMI men | rs2336030 | 1 | 11659187 | c | t | 0.65 | 1.35E-06 | *MAD2L2* |
| SAT men | rs7617219 | 3 | 150460004 | a | g | 0.60 | 1.38E-06 | *CP* |
| VATSAT women | rs12316797 | 12 | 41447011 | g | t | 0.13 | 1.39E-06 | *PRICKLE1* |
| SAT overall | rs11154271 | 6 | 125198148 | c | t | 0.22 | 1.39E-06 | *TCBA1* |
| SAT overall | rs869834 | 14 | 62246897 | t | c | 0.59 | 1.50E-06 | *KCNH5* |
| VATSAT overall | rs10089517 | 8 | 60341275 | c | a | 0.71 | 1.57E-06 | *TOX* |
| VATSAT women | rs7433808 | 3 | 64702126 | a | t | 0.75 | 1.60E-06 | *ADAMTS9* |
| VATSAT overall | rs4376189 | 4 | 186849669 | a | g | 0.90 | 1.61E-06 | *SORBS2* |
| VAT men | rs10910018 | 1 | 3641269 | a | g | 0.04 | 1.77E-06 | *KIAA0495;LRRC47;TP73;CCDC27* |
| VATSAT overall | rs7705033 | 5 | 122802684 | g | c | 0.72 | 1.80E-06 | *CCDC100* |
| VAT women | rs7627289 | 3 | 168831532 | a | g | 0.22 | 1.83E-06 | *WDR49* |
| VATaBMI women | rs9409154 | 9 | 117242537 | c | t | 0.12 | 1.87E-06 |  |
| VATaBMI women | rs12943829 | 17 | 68337999 | c | g | 0.01 | 1.91E-06 | *SLC39A11* |
| SAT overall | rs2324999 | 3 | 86241575 | t | c | 0.20 | 1.98E-06 | *CADM2* |
| VATSAT overall | rs17025426 | 1 | 110446850 | c | t | 0.94 | 2.01E-06 | *UBL4B;FAM40A;SLC6A17;ALX3* |
| SAT overall | rs17008402 | 3 | 71383257 | g | a | 0.31 | 2.03E-06 | *FOXP1* |
| VATSAT overall | rs10992471 | 9 | 94599326 | g | a | 0.57 | 2.03E-06 | *BICD2;ZNF484* |
| VATaBMI men | rs12175489 | 6 | 31485566 | a | g | 0.15 | 2.13E-06 | *HLA-B;HCP5* |
| VATSAT men | rs12562437 | 1 | 3640891 | t | c | 0.04 | 2.15E-06 | *KIAA0495;LRRC47;TP73;CCDC27* |
| VATSAT women | rs12022722 | 1 | 217717756 | t | c | 0.47 | 2.16E-06 | *LYPLAL1* |
| SAT overall | rs7245708 | 19 | 50887596 | c | a | 0.04 | 2.25E-06 | *QPCTL;FBXO46;EML2;SNRPD2;GIPR* |
| VAT women | rs3846635 | 5 | 82996490 | g | a | 0.08 | 2.37E-06 | *HAPLN1* |
| VATaBMI men | rs1502172 | 3 | 136718048 | a | g | 0.34 | 2.59E-06 | *EPHB1* |
| VATSAT men | rs8036080 | 15 | 38983676 | a | g | 0.01 | 2.65E-06 | *CHAC1;RHOV;DLL4;SPINT1;VPS18* |
| SAT women | rs12629805 | 3 | 148114854 | c | t | 0.10 | 2.68E-06 | *PLSCR5* |
| VATaBMI men | rs227458 | 6 | 165386341 | c | t | 0.87 | 2.72E-06 | *C6orf118* |
| VATSAT women | rs6789987 | 3 | 189246092 | c | t | 0.71 | 2.84E-06 | *LPP* |
| SAT men | rs1582861 | 2 | 137207591 | t | c | 0.50 | 2.87E-06 | *THSD7B* |
| VATaBMI men | rs31872 | 5 | 140352406 | g | a | 0.39 | 2.88E-06 | *PCDHAC1* |
| SAT women | rs2025934 | 1 | 192001523 | g | a | 0.93 | 2.89E-06 | *CDC73* |
| SAT women | rs1421084 | 16 | 52315241 | a | g | 0.97 | 3.00E-06 | *FTO* |
| VAT men | rs4701523 | 5 | 26174895 | t | a | 0.06 | 3.03E-06 | *CDH9* |
| VAT men | rs6876835 | 5 | 6652222 | a | g | 0.58 | 3.03E-06 | *NSUN2;SRD5A1* |
| VAT men | rs7294372 | 12 | 113121806 | t | c | 0.93 | 3.10E-06 | *TBX5* |
| VATSAT overall | rs10951138 | 7 | 26448329 | a | g | 0.18 | 3.14E-06 | *SNX10* |
| VATaBMI men | rs17216035 | 3 | 6456625 | a | c | 0.28 | 3.22E-06 | *GRM7* |
| VATSAT overall | rs10457442 | 6 | 98689018 | g | a | 0.93 | 3.28E-06 | *POU3F2* |
| VAT women | rs13323436 | 3 | 85585535 | a | t | 0.10 | 3.28E-06 | *CADM2* |
| SAT men | rs2173063 | 15 | 90932636 | a | g | 0.06 | 3.29E-06 | *LOC400451* |
| VATaBMI overall | rs6781182 | 3 | 34187170 | t | c | 0.29 | 3.31E-06 | *PDCD6IP* |
| VAT women | rs13196329 | 6 | 32433349 | c | a | 0.02 | 3.38E-06 | *C6orf10* |
| VAT women | rs815847 | 9 | 83412438 | g | a | 0.36 | 3.44E-06 | *TLE1* |
| VAT women | rs11839514 | 13 | 92736707 | g | a | 0.34 | 3.44E-06 | *GPC6* |
| SAT women | rs2324999 | 3 | 86241575 | t | c | 0.20 | 3.51E-06 | *CADM2* |
| VATSAT women | rs8013477 | 14 | 48604175 | c | g | 0.24 | 3.59E-06 | *RPS29* |
| VATSAT women | rs9899891 | 17 | 14570471 | a | g | 0.74 | 3.72E-06 | *FLJ45831* |
| VATSAT overall | rs6124878 | 20 | 44931130 | a | g | 0.06 | 3.74E-06 | *EYA2* |
| SAT men | rs990871 | 1 | 72596301 | t | c | 0.61 | 3.76E-06 | *NEGR1* |
| VATSAT men | rs10120372 | 9 | 107480363 | c | t | 0.06 | 3.98E-06 | *TAL2;FCMD;TMEM38B* |
| VAT overall | rs16910421 | 11 | 12027241 | a | g | 0.03 | 4.18E-06 | *DKK3* |
| VATSAT men | rs2943640 | 2 | 226801829 | c | a | 0.63 | 4.24E-06 | *IRS1* |
| VATaBMI men | rs1299548 | 7 | 7268818 | a | g | 0.46 | 4.25E-06 | *C1GALT1* |
| VATSAT overall | rs1056053 | 6 | 166491433 | t | c | 0.59 | 4.29E-06 | *T* |
| VAT women | rs1411916 | 9 | 80130396 | a | g | 0.11 | 4.29E-06 | *PSAT1* |
| SAT women | rs1453160 | 2 | 207585710 | a | g | 0.13 | 4.30E-06 | *CPO* |
| VATaBMI overall | rs2842895 | 6 | 7051315 | c | g | 0.58 | 4.32E-06 | *RREB1* |
| VATaBMI men | rs2287654 | 8 | 48066866 | t | g | 0.88 | 4.33E-06 | *KIAA0146* |
| VAT men | rs326155 | 5 | 7869645 | g | a | 0.01 | 4.39E-06 | *ADCY2* |
| VATSAT men | rs1536827 | 10 | 135156148 | c | t | 0.07 | 4.40E-06 | *CYP2E1* |
| VAT men | rs1530947 | 14 | 49021100 | t | c | 0.80 | 4.52E-06 | *RPS29* |
| SAT overall | rs5743030 | 2 | 190386790 | a | g | 0.05 | 4.54E-06 | *PMS1* |
| VAT women | rs11231299 | 11 | 62527964 | a | g | 0.20 | 4.58E-06 | *SLC22A8* |
| VATaBMI overall | rs1316952 | 12 | 122965503 | t | c | 0.87 | 4.60E-06 | *DNAH10* |
| VATaBMI women | rs11620399 | 13 | 47334926 | t | g | 0.04 | 4.60E-06 | *SUCLA2* |
| VATSAT men | rs8106493 | 19 | 38405768 | t | c | 0.20 | 4.64E-06 | *SLC7A10* |
| SAT men | rs6013355 | 20 | 50071067 | a | g | 0.84 | 4.80E-06 | *ZFP64* |
| VAT men | rs3819055 | 21 | 23711428 | g | c | 0.27 | 4.95E-06 | *NCAM2* |
| VATaBMI men | rs7525133 | 1 | 154608118 | a | g | 0.09 | 5.05E-06 | *RHBG* |
| VATSAT men | rs12636148 | 3 | 163614102 | c | t | 0.11 | 5.07E-06 | *LOC131149* |
| VATaBMI men | rs7120173 | 11 | 116334863 | g | t | 0.22 | 5.08E-06 | *KIAA0999* |
| VATSAT women | rs11620399 | 13 | 47334926 | t | g | 0.04 | 5.09E-06 | *SUCLA2* |
| VAT overall | rs10772915 | 12 | 16297957 | g | a | 0.57 | 5.34E-06 | *MGST1* |
| VAT overall | rs11683197 | 2 | 64881128 | t | c | 0.72 | 5.35E-06 | *SERTAD2* |
| VAT men | rs2278255 | 5 | 170653338 | g | c | 0.06 | 5.39E-06 | *RANBP17* |
| VATSAT overall | rs6577655 | 8 | 135662907 | t | c | 0.33 | 5.49E-06 | *ZFAT1* |
| SAT women | rs10916025 | 1 | 224922512 | g | c | 0.80 | 5.55E-06 | *ITPKB* |
| SAT women | rs11850957 | 14 | 24549382 | t | c | 0.21 | 5.63E-06 | *STXBP6* |
| VATaBMI men | rs9662633 | 1 | 3639422 | a | g | 0.05 | 5.64E-06 | *TP73* |
| SAT men | rs12583882 | 13 | 46471617 | g | a | 0.34 | 5.73E-06 | *HTR2A* |
| VAT men | rs10901513 | 10 | 127689926 | t | c | 0.60 | 5.90E-06 | *ADAM12;FANK1* |
| VATaBMI men | rs2059397 | 1 | 215784755 | g | c | 0.03 | 6.11E-06 | *GPATCH2* |
| VATaBMI overall | rs746080 | 16 | 82831906 | t | g | 0.31 | 6.21E-06 | *ADAD2;TAF1C;KCNG4;WFDC1* |
| VATaBMI overall | rs10914967 | 1 | 34761824 | a | g | 0.25 | 6.33E-06 | *GJB5* |
| VATaBMI women | rs4821132 | 22 | 31896587 | c | t | 0.56 | 6.35E-06 | *LARGE* |
| VATSAT overall | rs4978053 | 9 | 26198857 | c | g | 0.06 | 6.38E-06 | *TUSC1* |
| VAT men | rs17744121 | 21 | 29635469 | g | a | 0.07 | 6.45E-06 | *BACH1* |
| VAT men | rs7085142 | 10 | 122929364 | t | c | 0.37 | 6.46E-06 | *BRWD2* |
| VAT men | rs745978 | 5 | 15338414 | c | t | 0.00 | 6.50E-06 | *FBXL7* |
| VATSAT women | rs1048497 | 12 | 123065495 | g | a | 0.83 | 6.51E-06 | *ZNF664* |
| SAT women | rs12773846 | 10 | 126265904 | g | a | 0.70 | 6.61E-06 | *LHPP* |
| VAT overall | rs11161851 | 1 | 75071370 | t | c | 0.57 | 6.63E-06 | *TYW3* |
| VATSAT overall | rs16912285 | 11 | 24209104 | g | a | 0.10 | 6.76E-06 | *LUZP2* |
| VAT overall | rs11998649 | 8 | 22675496 | a | c | 0.19 | 6.77E-06 | *PEBP4* |
| SAT women | rs11952171 | 5 | 173628384 | g | a | 0.18 | 6.82E-06 | *HMP19* |
| SAT overall | rs2058059 | 7 | 71949753 | c | t | 0.24 | 6.88E-06 | *POM121* |
| VATSAT men | rs12486865 | 3 | 101439246 | g | c | 0.72 | 6.94E-06 | *TBC1D23;C3orf26* |
| VATaBMI men | rs7602441 | 2 | 14718952 | g | a | 0.88 | 6.98E-06 | *FAM84A* |
| VATaBMI men | rs10961577 | 9 | 14552314 | c | t | 0.96 | 7.02E-06 | *ZDHHC21* |
| VATaBMI overall | rs2554152 | 3 | 141089484 | g | t | 0.89 | 7.04E-06 | *CLSTN2* |
| SAT overall | rs12967884 | 18 | 74692623 | a | g | 0.89 | 7.11E-06 | *SALL3* |
| VAT men | rs10176755 | 2 | 171074135 | g | a | 0.00 | 7.25E-06 | *MYO3B* |
| VATaBMI men | rs13043330 | 20 | 3704597 | c | t | 0.63 | 7.25E-06 | *HSPA12B* |
| SAT overall | rs12467609 | 2 | 134859757 | t | c | 0.64 | 7.27E-06 | *MGAT5* |
| VATaBMI women | rs17302400 | 15 | 57612279 | t | c | 0.13 | 7.32E-06 | *FAM81A* |
| VAT women | rs10066447 | 5 | 13295974 | t | c | 0.23 | 7.43E-06 | *DNAH5* |
| VATaBMI women | rs6946494 | 7 | 135139161 | a | g | 0.83 | 7.54E-06 | *UNQ1940* |
| SAT women | rs13153333 | 5 | 91017143 | g | a | 0.04 | 7.57E-06 | *ARRDC3* |
| VATaBMI overall | rs4304868 | 12 | 116654292 | a | c | 0.16 | 7.57E-06 | *KSR2* |
| VAT overall | rs962528 | 2 | 6614539 | t | c | 0.32 | 7.60E-06 | *LOC129607* |
| VATaBMI men | rs7324557 | 13 | 23194862 | g | a | 0.65 | 7.68E-06 | *TNFRSF19;MIPEP* |
| VATaBMI overall | rs4657015 | 1 | 159539065 | g | a | 0.58 | 7.95E-06 | *LOC642502;PCP4L1;MPZ;SDHC* |
| VATSAT women | rs13177918 | 5 | 149806063 | t | c | 0.20 | 8.07E-06 | *RPS14* |
| SAT men | rs4886088 | 13 | 58026527 | a | g | 0.80 | 8.10E-06 | *PCDH17* |
| VAT overall | rs2267193 | 22 | 32076526 | c | g | 0.28 | 8.14E-06 | *LARGE* |
| VATSAT men | rs1791581 | 11 | 106001248 | a | g | 0.17 | 8.16E-06 | *GUCY1A2* |
| VAT women | rs6546102 | 2 | 64864060 | g | a | 0.51 | 8.18E-06 | *SERTAD2* |
| VATSAT men | rs1498095 | 3 | 42068426 | t | c | 0.91 | 8.21E-06 | *TRAK1* |
| VATaBMI women | rs7638389 | 3 | 64704932 | a | g | 0.74 | 8.23E-06 | *ADAMTS9* |
| VATSAT overall | rs7547921 | 1 | 38374660 | a | c | 0.09 | 8.23E-06 | *POU3F1* |
| VATaBMI overall | rs10516635 | 4 | 118299355 | a | g | 0.11 | 8.38E-06 | *TRAM1L1* |
| SAT overall | rs11757661 | 6 | 89240299 | a | g | 0.04 | 8.43E-06 | *RNGTT* |
| VATaBMI overall | rs1048497 | 12 | 123065495 | g | a | 0.83 | 8.51E-06 | *ZNF664* |
| VATaBMI women | rs2777777 | 9 | 83402980 | c | t | 0.55 | 8.63E-06 | *TLE1* |
| VAT men | rs6504663 | 17 | 45946208 | a | g | 0.76 | 8.63E-06 | *MYCBPAP* |
| VATSAT women | rs7042950 | 9 | 76339657 | a | g | 0.80 | 8.68E-06 | *RORB* |
| VAT women | rs7336109 | 13 | 105387142 | t | c | 0.29 | 8.71E-06 | *DAOA* |
| VAT men | rs2001970 | 16 | 53812692 | t | c | 0.51 | 8.79E-06 | *IRX6* |
| VATaBMI women | rs6789987 | 3 | 189246092 | c | t | 0.71 | 8.81E-06 | *LPP* |
| VAT men | rs17022027 | 4 | 95833301 | c | t | 0.94 | 8.92E-06 | *PDLIM5* |
| SAT overall | rs6686886 | 1 | 154623104 | g | a | 0.14 | 9.01E-06 | *CCT3;C1orf182;C1orf61;RHBG* |
| VAT men | rs9375969 | 6 | 133909495 | g | a | 0.76 | 9.18E-06 | *EYA4* |
| SAT women | rs870288 | 16 | 5525853 | a | g | 0.70 | 9.19E-06 | *FAM86A* |
| VAT men | rs12285276 | 11 | 45770157 | g | c | 0.76 | 9.22E-06 | *CRY2;SLC35C1* |
| VATSAT men | rs6464816 | 7 | 146857824 | a | g | 0.15 | 9.23E-06 | *CNTNAP2* |
| SAT overall | rs11858577 | 15 | 64853617 | t | c | 0.08 | 9.29E-06 | *SMAD6* |
| VATaBMI men | rs901254 | 5 | 178631917 | c | t | 0.96 | 9.30E-06 | *ADAMTS2* |
| VATSAT men | rs9861887 | 3 | 1320425 | t | c | 0.76 | 9.35E-06 | *CNTN6* |
| SAT women | rs12185578 | 2 | 17472720 | a | t | 0.23 | 9.42E-06 | *RAD51AP2* |
| VATSAT women | rs11190378 | 10 | 101789744 | t | c | 0.60 | 9.66E-06 | *CPN1;DNMBP* |
| VAT men | rs6013100 | 20 | 49203614 | t | c | 0.07 | 9.66E-06 | *KCNG1* |
| SAT men | rs967370 | 15 | 33254388 | g | t | 0.81 | 9.69E-06 | *ZNF770* |
| VAT men | rs17627810 | 1 | 214936637 | g | c | 0.08 | 9.70E-06 | *ESRRG* |
| VATSAT women | rs12800000 | 11 | 63970003 | g | t | 0.66 | 9.81E-06 | *RPS6KA4* |
| VATaBMI men | rs10895548 | 11 | 103325727 | a | g | 0.52 | 9.85E-06 | *PDGFD* |
| VATSAT men | rs4796409 | 17 | 7210097 | c | t | 0.40 | 9.86E-06 | *TNK1;EIF5A;PLSCR3;TMEM95* |
| VATaBMI men | rs1007381 | 14 | 23879451 | g | a | 0.43 | 9.93E-06 | *LTB4R* |
| VATSAT women | rs7904239 | 10 | 85569862 | a | g | 0.11 | 9.95E-06 | *GHITM* |
| VATaBMI women | rs1560742 | 12 | 71303657 | c | g | 0.83 | 9.97E-06 | *TRHDE* |
