# Supplementary material for: Genome-Wide Association for Abdominal Subcutaneous and Visceral Adipose Reveals a Novel Locus for Visceral Fat in Women
Source: PLoS Genet. 2012 May 10;8(5):e1002695. doi: 10.1371/journal.pgen.1002695 (PMC3349734; doi:10.1371/journal.pgen.1002695)
Supplement: Text S1 — Investigator names for the MAGIC Consortium. (DOC) [file pgen.1002695.s005.doc]

MAGIC Authors and Affiliations

Josée Dupuis^1,2,177^, Claudia Langenberg^3,177^, Inga Prokopenko^4,5,177^, Richa Saxena^6,7,177^, Nicole Soranzo^8,9,177^, Anne U Jackson^10^, Eleanor Wheeler^11^, Nicole LGlazer^12^, Nabila Bouatia-Naji^13^, Anna LGloyn^4^, Cecilia MLindgren^4,5^, Reedik Mägi^4,5^, Andrew P Morris^5^, Joshua Randall^5^, Toby Johnson^14–16^, Paul Elliott^17,176^, Denis Rybin^18^, Gudmar Thorleifsson^19^, Valgerdur Steinthorsdottir^19^, Peter Henneman^20^, Harald Grallert^21^, Abbas Dehghan^22^, Jouke Jan Hottenga^23^, Christopher SFranklin^24^, Pau Navarro^25^, Kijoung Song^26^, Anuj Goel^5,27^, John R B Perry^28^, Josephine MEgan^29^, Taina Lajunen^30^, Niels Grarup^31^, Thomas Sparsø^31^, Alex Doney^32^, Benjamin F Voight^6,7^, Heather MStringham^10^, Man Li^33^, Stavroula Kanoni^34^, Peter Shrader^35^, Christine Cavalcanti-Proença^13^, Meena Kumari^36^, Lu Qi^37^, Nicholas J Timpson^38^, Christian Gieger^21^, Carina Zabena^39^, Ghislain Rocheleau^40,41^, Erik Ingelsson^42,43^, Ping An^44^, Jeffrey O’Connell^45^, Jian’an Luan^3^, Amanda Elliott^6,7^, Steven A McCarroll^6,7^, Felicity Payne^11^, Rosa Maria Roccasecca^11^, François Pattou^46^, Praveen Sethupathy^47^, Kristin Ardlie^48^, Yavuz Ariyurek^49^, Beverley Balkau^50^, Philip Barter^51^, John P Beilby^52,53^, Yoav Ben-Shlomo^54^, Rafn Benediktsson^55,56^, Amanda J Bennett^4^, Sven Bergmann^14,16^, Murielle Bochud^15^, Eric Boerwinkle^57^, Amélie Bonnefond^13^, Lori LBonnycastle^47^, Knut Borch-Johnsen^58,59^, Yvonne Böttcher^60^, Eric Brunner^36^, Suzannah J Bumpstead^8^, Guillaume Charpentier^61^, Yii-Der Ida Chen^62^, Peter Chines^47^, Robert Clarke^63^, Lachlan J MCoin^17^, Matthew NCooper^64^, Marilyn Cornelis^37^, Gabe Crawford^6^, Laura Crisponi^65^, Ian NMDay^38^, Eco J Cde Geus^23^, Jerome Delplanque^13^, Christian Dina^13^, Michael R Erdos^47^, Annette CFedson^64,66^, Antje Fischer-Rosinsky^67,68^, Nita GForouhi^3^, Caroline SFox^2,69^, Rune Frants^70^, Maria Grazia Franzosi^71^, Pilar Galan^72^, Mark OGoodarzi^62^, Jürgen Graessler^73^, Christopher J Groves^4^, Scott Grundy^74^, Rhian Gwilliam^8^, Ulf Gyllensten^75^, Samy Hadjadj^76^, Göran Hallmans^77^, Naomi Hammond^8^, Xijing Han^10^, Anna-Liisa Hartikainen^78^, Neelam Hassanali^4^, Caroline Hayward^25^, Simon CHeath^79^, Serge Hercberg^80^, Christian Herder^81^, Andrew A Hicks^82^, David R Hillman^66,83^, Aroon DHingorani^36^, Albert Hofman^22^, Jennie Hui^52,84^, Joe Hung^85,86^, Bo Isomaa^87,88^, Paul R V Johnson^4,89^, Torben Jørgensen^90,91^, Antti Jula^92^, Marika Kaakinen^93^, Jaakko Kaprio^94–96^, Y Antero Kesaniemi^97^, Mika Kivimaki^36^, Beatrice Knight^98^, Seppo Koskinen^99^, Peter Kovacs^100^, Kirsten Ohm Kyvik^101^, GMark Lathrop^79^, Debbie A Lawlor^38^, Olivier Le Bacquer^13^, Cécile Lecoeur^13^, Yun Li^10^, Valeriya Lyssenko^102^, Robert Mahley^103^, Massimo Mangino^9^, Alisa KManning^1^, María Teresa Martínez-Larrad^39^, Jarred B McAteer^6,104,105^, Laura J McCulloch^4^, Ruth McPherson^106^, Christa Meisinger^21^, David Melzer^28^, David Meyre^13^, Braxton DMitchell^45^, Mario A Morken^47^, Sutapa Mukherjee^66,83^, Silvia Naitza^65^, Narisu Narisu^47^, Matthew J Neville^4,107^, Ben A Oostra^108^, Marco Orrù^65^, Ruth Pakyz^45^, Colin NA Palmer^109^, Giuseppe Paolisso^110^, Cristian Pattaro^82^, Daniel Pearson^47^, John F Peden^5,27^, Nancy LPedersen^42^, Markus Perola^96,111,112^, Andreas F H Pfeiffer^67,68^, Irene Pichler^82^, Ozren Polasek^113^, Danielle Posthuma^23,114^, Simon CPotter^8^, Anneli Pouta^115^, Michael A Province^44^, Bruce MPsaty^116,117^, Wolfgang Rathmann^118^, Nigel WRayner^4,5^, Kenneth Rice^119^, Samuli Ripatti^96,111^, Fernando Rivadeneira^22,120^, Michael Roden^81,121^, Olov Rolandsson^122^, Annelli Sandbaek^123^, Manjinder Sandhu^3,124^, Serena Sanna^65^, Avan Aihie Sayer^125^, Paul Scheet^126^, Laura J Scott^10^, Udo Seedorf^127^, Stephen J Sharp^3^, Beverley Shields^98^, Gunnar Sigurðsson^55,56^, Eric J GSijbrands^22,120^, Angela Silveira^128^, Laila Simpson^64,66^, Andrew Singleton^129^, Nicholas LSmith^130,131^, Ulla Sovio^17^, Amy Swift^47^, Holly Syddall^125^, Ann-Christine Syvänen^132^, Toshiko Tanaka^133,134^, Barbara Thorand^21^, Jean Tichet^135^, Anke Tönjes^60,136^, Tiinamaija Tuomi^87,137^, André GUitterlinden^22,120^, Ko Willems van Dijk^70,138^, Mandy van Hoek^120^, Dhiraj Varma^8^, Sophie Visvikis-Siest^139^, Veronique Vitart^25^, Nicole Vogelzangs^140^, Gérard Waeber^141^, Peter J Wagner^96,111^, Andrew Walley^142^, GBragi Walters^19^, Kim LWard^64,66^, Hugh Watkins^5,27^, Michael NWeedon^28^, Sarah H Wild^24^, Gonneke Willemsen^23^, Jaqueline CMWitteman^22^, John WGYarnell^143^, Eleftheria Zeggini^5,8^, Diana Zelenika^79^, Björn Zethelius^43,144^, Guangju Zhai^9^, Jing Hua Zhao^3^, MCarola Zillikens^120^, DIAGRAMConsortium^145^, GIANTConsortium^145^, Global BPgen Consortium^145^, Ingrid B Borecki^44^, Ruth J F Loos^3^, Pierre Meneton^80^, Patrik KEMagnusson^42^, David MNathan^104,105^, Gordon H Williams^69,105^, Andrew THattersley^98^, Kaisa Silander^96,111^, Veikko Salomaa^146^, George Davey Smith^38^, Stefan R Bornstein^73^, Peter Schwarz^73^, Joachim Spranger^67,68^, Fredrik Karpe^4,107^, Alan R Shuldiner^45^, Cyrus Cooper^125^, George V Dedoussis^34^, Manuel Serrano-Ríos^39^, Andrew DMorris^109^, Lars Lind^132^, Lyle J Palmer^64,66,84^, Frank B Hu1^47,148^, Paul WFranks^149^, Shah Ebrahim^150^, Michael Marmot^36^, WH Linda Kao^33,151,152^, James SPankow^153^, Michael J Sampson^154^, Johanna Kuusisto^155^, Markku Laakso^155^, Torben Hansen^31,156^, Oluf Pedersen^31,59,157^, Peter Paul Pramstaller^82,158,159^, H Erich Wichmann^21,160,161^, Thomas Illig^21^, Igor Rudan^24,162,163^, Alan F Wright^25^, Michael Stumvoll^60^, Harry Campbell^24^, James F Wilson^24^, Anders Hamsten on behalf of Procardis Consortium^128^, Richard NBergman^164^, Thomas A Buchanan^164,165^, Francis SCollins^47^, Karen LMohlke^166^, Jaakko Tuomilehto^94,167, 168^, Timo TValle^167^, David Altshuler^6,7,104,105^, Jerome I Rotter^62^, David SSiscovick^169^, Brenda WJ H Penninx^140^, Dorret I Boomsma^23^, Panos Deloukas^8^, Timothy DSpector^8,9^, Timothy MFrayling^28^, Luigi Ferrucci^170^, Augustine Kong^19^, Unnur Thorsteinsdottir^19,171^, Kari Stefansson^19,171^, Cornelia Mvan Duijn^22^, Yurii SAulchenko^22^, Antonio Cao^65^, Angelo Scuteri^172,177^, David Schlessinger^47^, Manuela Uda^65^, Aimo Ruokonen^173^, Marjo-Riitta Jarvelin^17,93, 174^, Dawn MWaterworth^26^, Peter Vollenweider^141^, Leena Peltonen^8,48,96,111,112^, Vincent Mooser^26^, Goncalo R Abecasis^10^, Nicholas J Wareham^3^, Robert Sladek^40,41^, Philippe Froguel^13,142^, Richard MWatanabe^164,175^, James B Meigs^35,105^, Leif Groop^102^, Michael Boehnke^10^, Mark I McCarthy^4,5,107^, Jose CFlorez^6,7,104,105^ & Inês Barroso^11^ for the MAGIC investigators

^1^Department of Biostatistics, Boston University School of Public Health, Boston, Massachusetts, USA. ^2^National Heart, Lung, and Blood Institute’s Framingham Heart Study, Framingham, Massachusetts, USA. ^3^Medical Research Council (MRC), Epidemiology Unit, Institute of Metabolic Science, Addenbrooke’s Hospital, Cambridge, UK. ^4^Oxford Centre for Diabetes, Endocrinology and Metabolism, University of Oxford, Oxford, UK. ^5^Wellcome Trust Centre for Human Genetics, University of Oxford, Oxford, UK. ^6^Program in Medical and Population Genetics, Broad Institute, Cambridge, Massachusetts, USA. ^7^Center for Human Genetic Research, Massachusetts General Hospital, Boston, Massachusetts, USA. ^8^Wellcome Trust Sanger Institute, Hinxton, Cambridge, UK. ^9^Twin Research and Genetic Epidemiology Department, King’s College London, St Thomas’ Hospital Campus, London, UK. ^10^Center for Statistical Genetics, Department of Biostatistics, University of Michigan School of Public Health, Ann Arbor, Michigan, USA. ^11^Metabolic Disease Group, Wellcome Trust Sanger Institute, Hinxton, Cambridge, UK. ^12^Cardiovascular Health Research Unit and Department of Medicine, University of Washington, Seattle, Washington, USA. ^13^Centre National de la Recherche Scientifique–Unité Mixte de Recherche 8090, Pasteur Institute, Lille 2–Droit et Santé University, Lille, France. ^14^Department of Medical Genetics, University of Lausanne, Lausanne, Switzerland. ^15^University Institute of Social and Preventative Medicine, Centre Hospitalier Universitaire Vaudois (CHUV) and University of Lausanne, Lausanne, Switzerland. ^16^Swiss Institute of Bioinformatics, Lausanne, Switzerland. ^17^Department of Epidemiology and Public Health, Imperial College London, Faculty of Medicine, Norfolk Place, London, UK. ^18^Boston University Data Coordinating Center, Boston, Massachusetts, USA. ^19^deCODE Genetics, Reykjavik, Iceland. ^20^Department of Human Genetics, Leiden University Medical Centre, Leiden, The Netherlands. ^21^Institute of Epidemiology, Helmholtz Zentrum Muenchen, German Research Center for Environmental Health, Neuherberg, Germany. ^22^Department of Epidemiology, Erasmus Medical College, Rotterdam, The Netherlands. ^23^Department of Biological Psychology, VU University Amsterdam, Amsterdam, The Netherlands. ^24^Centre for Population Health Sciences, University of Edinburgh, Edinburgh, UK. ^25^MRC Human Genetics Unit, Institute of Genetics and Molecular Medicine, Edinburgh, UK. ^26^Division of Genetics, Research and Development, GlaxoSmithKline, King of Prussia, Pennsylvania, USA. ^27^Department of Cardiovascular Medicine, University of Oxford, Oxford, UK. ^28^Genetics of Complex Traits, Institute of Biomedical and Clinical Sciences, Peninsula College of Medicine and Dentistry, University of Exeter, Exeter, UK. ^29^National Institute of Aging, Baltimore, Maryland, USA. ^30^Unit for Child and Adolescent Health and Welfare, National Institute for Health and Welfare, Biocenter Oulu, University of Oulu, Oulu, Finland. ^31^Hagedorn Research Institute, Gentofte, Denmark. ^32^Department of Medicine and Therapeutics, Level 7, Ninewells Hospital and Medical School, Dundee, UK. ^33^Department of Epidemiology, Bloomberg School of Public Health, Johns Hopkins University, Baltimore, Maryland, USA. ^34^Department of Nutrition–Dietetics, Harokopio University, Athens, Greece. ^35^General Medicine Division, Massachusetts General Hospital, Boston, Massachusetts, USA. ^36^Department of Epidemiology and Public Health, University College London, London, UK. ^37^Departments of Nutrition and Epidemiology, Harvard School of Public Health, Boston, Massachusetts, USA. ^38^MRC Centre for Causal Analyses in Translational Epidemiology, University of Bristol, Bristol, UK. ^39^Fundación para la Investigación Biomédica del Hospital Clínico San Carlos, Madrid, Spain. ^40^Departments of Medicine and Human Genetics, McGill University, Montreal, Canada. ^41^Genome Quebec Innovation Centre, Montreal, Canada. ^42^Department of Medical Epidemiology and Biostatistics, Karolinska Institutet, Stockholm, Sweden. ^43^Department of Public Health and Caring Sciences, Uppsala University, Uppsala, Sweden. ^44^Division of Statistical Genomics, Department of Genetics, Washington University School of Medicine, St. Louis, Missouri, USA. ^45^Division of Endocrinology, Diabetes and Nutrition, University of Maryland School of Medicine, Baltimore, Maryland, USA. ^46^INSERM U859, Universite de Lille-Nord de France, Lille, France. ^47^Genome Technology Branch, National Human Genome Research Institute, Bethesda, Maryland, USA. ^48^The Broad Institute, Cambridge, Massachusetts, USA. ^49^Leiden Genome Technology Center, Leiden University Medical Center, Leiden, The Netherlands. ^50^INSERM U780, Paris Sud University, Villejuif, France. ^51^The Heart Research Institute, Sydney, New South Wales, Australia. ^52^PathWest Laboratory of Western Australia, Department of Molecular Genetics, J Block, QEII Medical Centre, Nedlands West Australia, Australia. ^53^School of Surgery and Pathology, University of Western Australia, Nedlands West Australia, Australia. ^54^Department of Social Medicine, University of Bristol, Bristol, UK. ^55^Landspitali University Hospital, Reykjavik, Iceland. ^56^Icelandic Heart Association, Kopavogur, Iceland. ^57^The Human Genetics Center and Institute of Molecular Medicine, University of Texas Health Science Center, Houston, Texas, USA. ^58^Steno Diabetes Center, Gentofte, Denmark. ^59^Faculty of Health Science, University of Aarhus, Aarhus, Denmark. ^60^Department of Medicine, University of Leipzig, Leipzig, Germany. ^61^Endocrinology–Diabetology Unit, Corbeil-Essonnes Hospital, Essonnes, France. ^62^Medical Genetics Institute, Cedars-Sinai Medical Center, Los Angeles, California, USA. ^63^Clinical Trial Service Unit and Epidemiological Studies Unit, University of Oxford, Oxford, UK. ^64^Centre for Genetic Epidemiology and Biostatistics, University of Western Australia, Perth, Australia. ^65^Istituto di Neurogenetica e Neurofarmacologia (INN), Consiglio Nazionale delle Ricerche, c/o Cittadella Universitaria di Monserrato, Monserrato, Cagliari, Italy. ^66^Western Australian Sleep Disorders Research Institute, Queen Elizabeth Medical Centre II, Perth, Australia. ^67^Department of Endocrinology, Diabetes and Nutrition, Charite-Universitaetsmedizin Berlin, Berlin, Germany. ^68^Department of Clinical Nutrition, German Institute of Human Nutrition Potsdam-Rehbruecke, Nuthetal, Germany. ^69^Division of Endocrinology, Diabetes, and Hypertension, Brigham and Women’s Hospital, Harvard Medical School, Boston, Massachusetts, USA. ^70^Department of Human Genetics, Leiden University Medical Centre, Leiden, The Netherlands. ^71^Department of Cardiovascular Research, Istituto di Ricerche Farmacologiche ‘Mario Negri’, Milan, Italy. ^72^Institut National de la Santé et de la Recherche Médicale, Institut National de la Recherche Agronomique, Université Paris 13, Bobigny Cedex, France. ^73^Department of Medicine III, Division Prevention and Care of Diabetes, University of Dresden, Dresden, Germany. ^74^Center for Human Nutrition, University of Texas Southwestern Medical Center, Dallas, Texas, USA. ^75^Department of Genetics and Pathology, Rudbeck Laboratory, Uppsala University, Uppsala, Sweden. ^76^Centre Hospitalier Universitaire, de Poitiers, Endocrinologie Diabetologie, CIC INSERM 0802, INSERM U927, Université de Poitiers, Unité de Formation et de Recherche, Médecine Pharmacie, Poitiers, France. ^77^Department of Public Health and Clinical Medicine, Section for Nutritional Research, Umeå University, Umeå, Sweden. ^78^Department of Clinical Sciences, Obstetrics and Gynecology, University of Oulu, University of Oulu, Finland. ^79^Centre National de Génotypage/Institut de génomique/Commissariat à l’énergie atomique, Evry Cedex, France. ^80^INSERM U872, Faculté de Médecine Paris Descartes, Paris Cedex, France. ^81^Institute for Clinical Diabetology, German Diabetes Center, Leibniz Center for Diabetes Research at Heinrich Heine University Düsseldorf, Düsseldorf, Germany. ^82^Institute of Genetic Medicine, European Academy Bozen/Bolzano (EURAC), Viale Druso, Bolzano, Italy, Affiliated Institute of the University Lübeck, Lübeck, Germany. ^83^Department of Pulmonary Physiology, Sir Charles Gairdner Hospital, Perth, Australia. ^84^Busselton Population Medical Research Foundation, Sir Charles Gairdner Hospital, Perth, Australia. ^85^Heart Institute of Western Australia, Sir Charles Gairdner Hospital, Nedlands West Australia, Australia. ^86^School of Medicine and Pharmacology, University of Western Australia, Nedlands West Australia, Australia. ^87^Folkhalsan Research Centre, Helsinki, Finland. ^88^Malmska Municipal Health Care Center and Hospital, Jakobstad, Finland. ^89^Nuffield Department of Surgery, University of Oxford, Oxford, UK. ^90^Research Centre for Prevention and Health, Glostrup University Hospital, Glostrup, Denmark. ^91^Faculty of Health Science, University of Copenhagen, Copenhagen, Denmark. ^92^National Institute for Health and Welfare, Unit of Population Studies, Turku, Finland. ^93^Institute of Health Sciences and Biocenter Oulu, University of Oulu, Oulu, Finland. ^94^Department of Public Health, Faculty of Medicine, University of Helsinki, Helsinki, Finland. ^95^National Institute for Health and Welfare, Unit for Child and Adolescent Mental Health, Helsinki, Finland. ^96^Institute for Molecular Medicine Finland (FIMM), University of Helsinki, Helsinki, Finland. ^97^Department of Internal Medicine and Biocenter Oulu, Oulu, Finland. ^98^Diabetes Genetics, Institute of Biomedical and Clinical Science, Peninsula College of Medicine and Dentistry, University of Exeter, Exeter, UK. ^99^National Institute for Health and Welfare, Unit of Living Conditions, Health and Wellbeing, Helsinki, Finland. ^100^Interdisciplinary Centre for Clinical Research, University of Leipzig, Leipzig, Germany. ^101^The Danish Twin Registry, Epidemiology, Institute of Public Health, University of Southern Denmark, Odense, Denmark. ^102^Department of Clinical Sciences, Diabetes and Endocrinology, Lund University, University Hospital Malmö, Malmö, Sweden. ^103^Gladstone Institute of Cardiovascular Disease, University of California, San Francisco, California, USA. ^104^Diabetes Research Center, Diabetes Unit, Massachusetts General Hospital, Boston, Massachusetts, USA. ^105^Department of Medicine, Harvard Medical School, Boston, Massachusetts, USA. ^106^Division of Cardiology, University of Ottawa Heart Institute, Ottawa, Ontario, Canada. ^107^Oxford National Institute for Health Research, Biomedical Research Centre, Churchill Hospital, Oxford, UK. ^108^Department of Clinical Genetics, Erasmus Medical College, Rotterdam, The Netherlands. ^109^Biomedical Research Institute, University of Dundee, Ninewells Hospital and Medical School, Dundee, UK. ^110^Department of Geriatric Medicine and Metabolic Disease, Second University of Naples, Naples, Italy. ^111^National Institute for Health and Welfare, Unit of Public Health Genomics, Helsinki, Finland. ^112^Department of Medical Genetics, University of Helsinki, Helsinki, Finland. ^113^Department of Medical Statistics, Epidemiology and Medical Informatics, Andrija Stampar School of Public Health, Medical School, University of Zagreb, Rockefellerova, Zagreb, Croatia. ^114^Department of Clinical Genetics, VU University and Medical Center, Amsterdam, The Netherlands. ^115^Department of Obstetrics and Gynaecology, Oulu University Hospital, Oulu, Finland. ^116^Departments of Medicine, Epidemiology and Health Services, University of Washington, Seattle, Washington, USA. ^117^Group Health Research Institute, Group Health Cooperative, Seattle, Washington, USA. ^118^Institute of Biometrics and Epidemiology, German Diabetes Centre, Leibniz Centre at Heinrich Heine University Düsseldorf, Düsseldorf, Germany. ^119^Department of Biostatistics, University of Washington, Seattle, Washington, USA. ^120^Department of Internal Medicine, Erasmus Medical College, Rotterdam, The Netherlands. ^121^Department of Metabolic Diseases, Heinrich Heine University Düsseldorf, Düsseldorf, Germany. ^122^Department of Public Health and Clinical Medicine, Section for Family Medicine, Umeå University, Umeå, Sweden. ^123^School of Public Health, Department of General Practice, University of Aarhus, Aarhus, Denmark. ^124^Department of Public Health and Primary Care, Strangeways Research Laboratory, University of Cambridge, Cambridge, UK. ^125^MRC Epidemiology Resource Centre, University of Southampton, Southampton General Hospital, Southampton, UK. ^126^Department of Epidemiology, University of Texas, M.D. Anderson Cancer Center, Houston, Texas, USA. ^127^Leibniz-Institut für Arterioskleroseforschung an der Universität Münster, Münster, Germany. ^128^Atherosclerosis Research Unit, Department of Medicine, Karolinska Institutet, Stockholm, Sweden. ^129^Laboratory of Neurogenetics, National Institute on Aging, Bethesda, Maryland, USA. ^130^Department of Epidemiology, University of Washington, Seattle, Washington, USA. ^131^Seattle Epidemiologic Research and Information Center, Department of Veterans Affairs Office of Research and Development, Seattle, Washington, USA. ^132^Department of Medical Sciences, Uppsala University, Uppsala, Sweden. ^133^Medstar Research Institute, Baltimore, Maryland, USA. ^134^Clinical Research Branch, National Institute on Aging, Baltimore, Maryland, USA. ^135^Institut interrégional pour la santé (IRSA), La Riche, France. ^136^Coordination Centre for Clinical Trials, University of Leipzig, Leipzig, Germany. ^137^Department of Medicine, Helsinki University Hospital, University of Helsinki, Helsinki, Finland. ^138^Department of Internal Medicine, Leiden University Medical Centre, Leiden, The Netherlands. ^139^Research Unit, Cardiovascular Genetics, Nancy University Henri Poincaré, Nancy, France. ^140^EMGO Institute for Health and Care Research, Department of Psychiatry, VU University Medical Center, Amsterdam, The Netherlands. ^141^Department of Internal Medicine, Centre Hospitalier Universitaire Vaudois, Lausanne, Switzerland. ^142^Genomic Medicine, Imperial College London, Hammersmith Hospital, London, UK. ^143^Epidemiology and Public Health, Queen’s University Belfast, Belfast, UK. ^144^Medical Products Agency, Uppsala, Sweden. ^145^See Supplementary Note for a full list of authors. ^146^National Institute for Health and Welfare, Unit of Chronic Disease Epidemiology and Prevention, Helsinki, Finland. ^147^Departments of Nutrition and Epidemiology, Harvard School of Public Health, Boston, Massachusetts, USA. ^148^Channing Laboratory, Brigham and Women’s Hospital and Harvard Medical School, Boston, Massachusetts, USA. ^149^Genetic Epidemiology and Clinical Research Group, Department of Public Health and Clinical Medicine, Section for Medicine, Umeå University Hospital, Umeå, Sweden. ^150^London School of Hygiene and Tropical Medicine, London, UK. ^151^Department of Medicine, School of Medicine, Johns Hopkins University, Baltimore, Maryland, USA. ^152^The Welch Center for Prevention, Epidemiology, and Clinical Research, School of Medicine and Bloomberg School of Public Health, Johns Hopkins University, Baltimore, Maryland, USA. ^153^Division of Epidemiology and Community Health, School of Public Health, University of Minnesota, Minneapolis, Minnesota, USA. ^154^Department of Endocrinology and Diabetes, Norfolk and Norwich University Hospital National Health Service Trust, Norwich, UK. ^155^Department of Medicine, University of Kuopio and Kuopio University Hospital, Kuopio, Finland. ^156^Faculty of Health Science, University of Southern Denmark, Odense, Denmark. ^157^Institute of Biomedical Science, Faculty of Health Science, University of Copenhagen, Copenhagen, Denmark. ^158^Department of Neurology, General Central Hospital, Bolzano, Italy. ^159^Department of Neurology, University of Lübeck, Lübeck, Germany. ^160^Institute of Medical Informatics, Biometry and Epidemiology, Ludwig-Maximilians-Universität, Munich, Germany. ^161^Klinikum Grosshadern, Munich, Germany. ^162^School of Medicine, University of Split, Split, Croatia. ^163^Gen-Info Ltd., Zagreb, Croatia. ^164^Department of Physiology and Biophysics, Keck School of Medicine, University of Southern California, Los Angeles, California, USA. ^165^Department of Medicine, Division of Endocrinology, Keck School of Medicine, University of Southern California, Los Angeles, California, USA. ^166^Department of Genetics, University of North Carolina, Chapel Hill, North Carolina, USA. ^167^National Institute for Health and Welfare, Unit of Diabetes Prevention, Helsinki, Finland. ^168^South Ostrobothnia Central Hospital, Seinajoki, Finland. ^169^Departments of Medicine and Epidemiology, University of Washington, Seattle, Washington, USA. ^170^Longitudinal Studies Section, Clinical Research Branch, National Institute on Aging, NIH, Baltimore, Maryland, USA. ^171^Faculty of Medicine, University of Iceland, Reykjavík, Iceland. ^172^Lab of Cardiovascular Sciences, National Institute on Aging, National Institutes of Health, Baltimore, Maryland, USA. ^173^Department of Clinical Sciences/Clinical Chemistry, University of Oulu, University of Oulu, Oulu, Finland. ^174^National Institute of Health and Welfare, Oulu, Finland. ^175^Department of Preventive Medicine, Keck School of Medicine, University of Southern California, Los Angeles, California, USA. ^176^MRC–Health Protection Agency Centre for Environment and Health, Imperial College London, London, UK. ^177^UOC Geriatria, Istituto Nazionale Ricovero e cura per Anziani (INRCA) IRCCS, Rome, Italy. ^178^These authors contributed equally to this work.
